# Gametic selection favours polyandry and selfing

**DOI:** 10.1371/journal.pgen.1010660

**Published:** 2024-02-16

**Authors:** Michael Francis Scott, Carl Mackintosh, Simone Immler

**Affiliations:** 1 School of Biological Sciences, University of East Anglia, Norwich, Norfolk, United Kingdom; 2 CNRS, UMR7144 Adaptation et Diversité en Milieu Marin, Station Biologique de Roscoff, Roscoff, France; 3 Sorbonne Universités, UPMC Université Paris VI, Roscoff, France; Stockholm University, SWEDEN

## Abstract

Competition among pollen or sperm (gametic selection) can cause evolution. Mating systems shape the intensity of gametic selection by determining the competitors involved, which can in turn cause the mating system itself to evolve. We model the bidirectional relationship between gametic selection and mating systems, focusing on variation in female mating frequency (monandry-polyandry) and self-fertilisation (selfing-outcrossing). First, we find that monandry and selfing both reduce the efficiency of gametic selection in removing deleterious alleles. This means that selfing can increase mutation load, in contrast to cases without gametic selection where selfing purges deleterious mutations and decreases mutation load. Second, we explore how mating systems evolve via their effect on gametic selection. By manipulating gametic selection, polyandry can evolve to increase the fitness of the offspring produced. However, this indirect advantage of post-copulatory sexual selection is weak and is likely to be overwhelmed by any direct fitness effects of mating systems. Nevertheless, gametic selection can be potentially decisive for selfing evolution because it significantly reduces inbreeding depression, which favours selfing. Thus, the presence of gametic selection could be a key factor driving selfing evolution.

## Author summary

Animals and plants often produce millions of sperm or pollen, of which only a tiny fraction succeed. This means sperm and pollen harbour a lot of evolutionary potential, even though they are generally small and hidden from view. We used mathematical models to understand the evolution of genes that influence the fertilisation success rate of sperm or pollen. A crucial issue is the number of different males who donate sperm or pollen, which is called the mating system. We can predict how genes will evolve with different mating systems. These predictions can then be tested by looking at DNA sequences from genes that are active in sperm or pollen in species with different mating systems. This process allows us to check our understanding of evolution. Secondly, we looked at the evolution of the mating behaviour itself. By changing their mating behaviour, animals and plants can manipulate natural selection on sperm or pollen, thereby evolving to change the way evolution operates. This helps to answer a bigger evolutionary question: why do organisms evolve differences in the way they respond to natural selection?

## Introduction

Males typically produce a large number of gametes or gametophytes (hereafter male gametes) that then compete to fertilise a small number of eggs or ovules (hereafter female gametes) [1] generating considerable selective pressure (hereafter gametic selection). The pool of male gametes that compete against each another depends on ‘who mates with whom’, which is called the mating system. Two important aspects of mating system variation are the number of males that females mate with [2] and the rate of self fertilisation [3]. That is, mating systems vary from monandry (females mate with one male) to polyandry (females mate with several males) and from selfing (where male and female gametes are derived from one individual) to outcrossing (where male and female gametes are derived from different individuals). Both axes of mating system variation affect the genetic composition of male gamete pools and with that gametic selection. We model interactions between mating systems and gametic selection from two angles, one to study the influence of gametic selection and mating systems on allele frequency dynamics and one to study the evolution of mating systems with gametic selection.

Evolutionary responses to gametic selection depend on the way genetic material is expressed, with significant variation across genes and taxa. Fertilisation success may depend on a gamete’s haploid genotype or the diploid genotype of the male that produced them. In flowering plants, 60–70% of all genes are expressed in haploid male gametophytes [4, 5] such that the fertilisation success of pollen is thought to depend partly on its haploid genotype [6, 7]. Indeed, haploid expression and pollen competition has been shown to cause non-random inheritance of genotypes from a single male [8–12] and pollen-expressed genes show stronger signatures of selection than random genes [13]. In animals, success during sperm competition is usually assumed to depend on the father’s diploid genotype [14]. This assumption is based on the cytoplasmic bridges that link developing spermatids, allowing transcript sharing and effectively diploid expression at most genes [15]. Nevertheless, recent results suggest that haploid expression and selection in animal sperm has been underestimated (reviewed in [16–18]). For example, sperm selection assays within single ejaculates of the zebrafish *Danio rerio* have been shown to cause allelic biases [19]. Single cell expression data from primate testes has revealed extensive expression at late stages of spermatogenesis, with these genes experiencing accelerated evolutionary rates [20]. Single cell expression is biased towards a haploid allele at 31–52% of spermatid-expressed genes in a range of mammals [21], i.e., approximately 20% of all genes. These results suggest that expression of different genes in animal sperm can vary continuously from haploid to diploid depending on the degree of allelic bias [22]. Our models allow a range of allelic bias scenarios to compare the effect of gametic selection across genes and taxa.

It is not straightforward to predict how mating systems and gametic selection interact to produce evolutionary responses. First, males produce gamete genotypes in equal proportions, maximising the genetic variation at heterozygous loci, which maximises the potential responses to selection. Thus, monandrous matings could plausibly increase gametic selection, as long as gametic expression is haploid. Diploid gametic expression, on the other hand, will eliminate fitness variation among gametes from a single male and prevent gametic selection under monandry. Second, haploid expression and selfing both affect the efficiency of purifying selection and the associated mutation load. In diploid heterozygotes, a homologous gene copy can (partially) mask the effects of deleterious alleles, preventing them from being efficiently removed by selection [23, 24]. Haploid expression means alleles are exposed to selection and selfing reduces masking by increasing homozygosity so both can reduce mutation load [23–27]. However, this effect is reversed when some alleles are favoured during gametic selection but reduce the fitness of diploid adults [28–30]. Under such a scenario, adult fitness would be optimised with less gametic selection.

The evolutionary responses to the interaction between mating systems and gametic selection is therefore rather complex and models are a useful way to examine these interactions. A previous model assuming haploid expression compared the expected mutation load between selfers and outcrossers [31, 32], and another theoretical study assessed the evolutionary outcome across selfing rates where gametes and adults have opposing selection pressures [33]. Most models of sperm competition assumed diploid control over sperm competition success, which means that gametic selection only occurs under polyandry (reviewed in [34, 35]). Nevertheless, two theoretical studies have examined genes with haploid expression in sperm and no adult effect, finding that haploid expression can allow evolution under monandry [36] and that evolutionary rates increase with haploid expression and the harmonic mean of the number of mates per female [37]. The most relevant study to consider the evolution of mating systems via their effect on gametic selection examines the ‘good sperm’ hypothesis using a quantitative genetics framework [38], finding that polyandry can evolve if mutations reduce viability and viability is positively correlated with sperm competitiveness. In our population genetic approach, the correlation between viability and gametic selection arises from selection at each locus and the ‘good sperm’ effect can be expressed with parameters such as the genome-wide deleterious mutation rate [39]. By considering a range of mating systems and expression patterns, we offer comparative predictions for empirical testing and contrast the evolutionary forces on mating system evolution.

We model the two-way interaction between gametic selection and mating systems. We first consider the influence of mating systems (monandry/ polyandry and selfing/outcrossing) and gametic expression patterns (ranging from haploid to diploid) on the evolution of alleles that affect fertilisation success. We then examine how gametic selection influences mating system evolution. We find that monandry and selfing both reduce the efficiency of gametic selection, but only monandry/polyandry can evolve via this effect because selfing also increases homozygosity. Nevertheless, gametic selection can substantially reduce inbreeding depression and thereby favour selfing.

## Methods

We investigate how allele frequencies change under different mating systems and the evolution of mating systems themselves. We construct two related models corresponding to two mating system scenarios: (a) mating systems fall on a spectrum from polyandrous to monandrous and (b) mating systems can vary from selfing to outcrossing. We are specifically interested in the effects of competition among male gametes. Here, we outline the key features of our model. We provide a detailed model description in S1 Appendix and S1 File, which can be used to replicate the results (see also S2 File). For reference, all the parameters and compound parameters used throughout the model and results are summarised in Table 1.

**Table 1:** Definitions of parameters and compound parameters.

Parameter	Description
**A**	locus experiencing selection with alleles *A* and *a*
**M**	modifier locus with alleles *M* and *m* that control mating system
*r*	recombination rate between **A** and **M** loci
$s_{g}^{h}$	diploid fitness of genotype *g* ∈ {*AA*, *Aa*, *aa*} as sex *h* ∈ {♂, ♀}, see Table 2
$s_{\alpha }^{g}$	gametic fitness of genotype *α* ∈ {*A*, *a*} produced by males of genotypes *g*, see Table 2
$s_{\Delta A}^{Aa}=-s_{\Delta a}^{Aa}$	fitness difference between *A*-bearing and *a*-bearing gametes from *Aa* males, $s_{\Delta A}^{Aa}=(s_{A}^{Aa}-s_{a}^{Aa})/2$
${\bar{s}}^{Aa}$	average fitness of gametes from *Aa* males, ${\bar{s}}^{Aa}=(s_{A}^{Aa}+s_{a}^{Aa})/2$
*σ*	strength of gametic selection against *a* allele, see Table 2
*γ*	function describing relationship between gametic fitness and *a* allele expression
*d*	gametic expression pattern varying from haploid (*d* = 0) to diploid (*d* = 1), see Fig 1a
*H*	dominance of *A* allele for gametic fitness
${\bar{s}}_{\alpha }^{♀}$	net selection in females for/against allele *α* ∈ {*A*, *a*} when rare, see Table 3
${\bar{s}}_{\alpha }^{♂,d}$	net selection in diploid males for/against allele *α* ∈ {*A*, *a*} when rare, see Table 3
${\bar{s}}_{\alpha }^{♂,g}$	net selection in male gametes for/against allele *α* ∈ {*A*, *a*} when rare, see Table 3
*I*_*α*_	factor determining spread of allele *α* ∈ {*A*, *a*} when rare, see Eq (1)
Π	proportion of polyandry versus monandry in a population with genotype *MM*
Π_*f*_	proportion of polyandry versus monandry for carriers of the rare modifier *f* ∈ {*Mm*, *mm*}
Δ_Π_	increase in polyandry caused by the modifier, Δ_Π_ = (Π_*Mm*_ − Π)/2
Ω	proportion of outcrossing versus selfing in a population with genotype *MM*
Ω_*f*_	proportion of outcrossing versus selfing for carriers of the rare modifier *f* ∈ {*Mm*, *mm*}
Δ_Ω_	increase in outcrossing caused by the modifier, Δ_Ω_ = (1 − *F*)(Ω_*Mm*_ − Ω) + *F*(Ω_*mm*_ − Ω)
*F*	inbreeding parameter describing excess homozygosity, *F* = (1 − Ω)/(1 + Ω) to leading order
*c*	relative reduction in male gametes available outcrossing due to selfing, a.k.a. pollen discounting
*μ*	per-locus mutation rate
${\hat{q}}_{\mu }$	equilibrium frequency of deleterious alleles at mutation-selection balance, see Eq (2)
${\hat{q}}_{B}$	equilibrium frequency alleles maintained by balancing selection, see Eq (3)
$\lambda_{\mu }^{\Pi }$	spread of rare polyandry/monandry modifier when the selected **A** locus is at mutation-selection balance
$\lambda_{B}^{\Pi }$	spread of rare polyandry/monandry modifier when the selected **A** locus is under balancing selection
$\lambda_{\mu }^{\Omega }$	spread of rare outcrossing/selfing modifier when the selected **A** locus is at mutation-selection balance
$\lambda_{\mu }^{\Pi }$	spread of rare outcrossing/selfing modifier when the selected **A** locus is under balancing selection
*l*_*B*_	number of loci experiencing balancing selection
*l*_*μ*_	number of loci at mutation-selection balance
*k*	proportion of loci at mutation-selection balance that experience gametic selection
*U*	haploid genome wide mutation rate, *U* = *μl*_*μ*_
*s*_*tot*_	combined selection on a modifier across loci
*δ*	inbreeding depression caused by a single locus
$\bar{\delta }$	genome-wide inbreeding depression

Primarily, we use a two-locus model with a fitness locus (**A**) that experiences selection directly and a modifier locus (**M**) that determines the mating system. The results from the two-locus model are then extrapolated to multiple loci and the accuracy of this extrapolation is compared against simulations. In the two-locus model, the alleles (*A* and *a*) at the fitness locus can have different effects on the fitness of adults of both sexes (fitness coefficients can differ between male and female adults) and of male gametes; all the fitness terms are given in Table 2. In hermaphrodites, alleles can differentially affect male and female fecundity [40] and therefore we also allow for separate male and female fitness effects in hermaphroditic selfers. The choice of mate is assumed to be independent of the **A** and **M** locus genotypes so we only model sexual selection that is post-copulatory and caused by gametic selection among male gametes.

**Table 2:** Fitness parameters for different life cycle stages.

stage	sex	genotype	fitness
adult	female	*AA*	$1+s_{AA}^{♀}$
		*Aa*	$1+s_{Aa}^{♀}$
		*aa*	$1+s_{aa}^{♀}$
	male	*AA*	$1+s_{AA}^{♂}$
		*Aa*	$1+s_{Aa}^{♂}$
		*aa*	$1+s_{aa}^{♂}$
gamete	from *AA* male	*A*	$1+s_{A}^{AA}=1$
	from *Aa* male*	*A*	$1+s_{A}^{Aa}=1-\gamma \left(d/2\right)\sigma$
		*a*	$1+s_{a}^{Aa}=1-\gamma \left(1-d/2\right)\sigma$
	from *aa* male	*a*	$1+s_{a}^{aa}=1-\sigma$

* *γ*(*x*) = 1 − (1 − *x*^*H*^)^1/*H*^ describes the relationship between fitness and *a*-allele expression where *H* is the gametic dominance of the *A* allele, *d* = 0 corresponds to haploid gametic expression, and *d* = 1 corresponds to diploid gametic expression.

**Fig 1:**
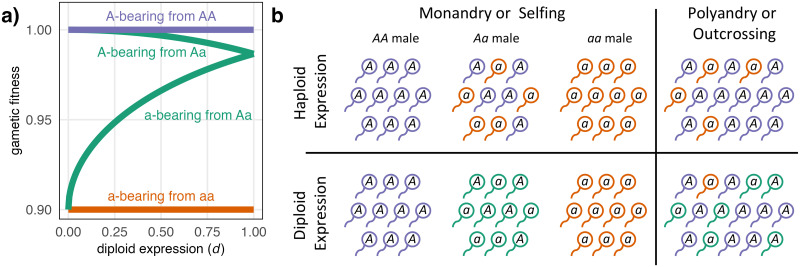
Illustration of competition among male gametes. (a) Gametic expression can vary continuously from fully haploid to fully diploid, with intermediate expression between. Here, the *a* allele is deleterious (*σ* = 0.1) and partially recessive (*H* = 2). (b) Male gametes compete in local pools according to the mating system (colours represent gametic fitness). When expression is fully haploid, heterozygous males create highly competitive gamete pools where allele frequencies are equal, but there is no fitness variation with diploid expression. Monandry involves outcrossing between male and female individuals whereas selfing involves the male and female gametes from the same individual. The frequency of the different male gamete pools under monandry/selfing and the allele frequency in the polyandry/outcrossing gamete pool depends on the population genotype frequency.

Male gametic fitness depends on the expression of *A* versus *a* alleles. That is, *A*-bearing gametes from *Aa* males can express their own genotype (100% *A* allele), or their father’s genotype (50% *A* alleles), or some combination. In general, we can account for any gametic expression pattern by using different selection coefficients for the four different combinations of gamete genotype and paternal genotype, as shown in Table 2. When gametic expression is haploid (*d* = 0), gametic fitness is independent of the paternal genotype so there are only two fitness levels. When gametic expression is diploid (*d* = 1), the gametes have one of three fitness levels depending on the diploid genotype of the father that produced them. We also allow intermediate gametic expression patterns [22], in which case we must specify the relationship between fitness and the proportion of *A*- versus *a*-allele expression, which we assume depends on the function *γ*(*x*) = 1 − (1 − *x*^*H*^)^1/*H*^. In our numerical results, we assume that the higher fitness *A* allele is dominant, with *H* = 2, so that increasingly diploid-like expression masks the deleterious effects of the *a* allele. Specifically, we assume that *AA* males produce *A*-bearing gametes with relative fitness 1 ($s_{A}^{AA}=0$) and *aa* males produce *a*-bearing gametes with fitness 1 − *σ* ($s_{a}^{aa}=-\sigma$, with 0 < *σ* < 1 such that the *a* allele is disfavoured during gametic competition). *Aa* males produce *A*-bearing gametes with fitness 1 − *γ*(*d*/2)*σ* and *a*-bearing gametes have fitness 1 − *γ*(1 − *d*/2)*σ* so that the fitness of both gamete types from heterozygous males is the same when expression is diploid (*d* = 1). Fig 1a shows an example of the relationship between allelic expression and male gametic fitness.

The way male gametes compete with each another is determined by the mating system (Fig 1b). Mating systems can be a mixture of (i) polyandry and monandry or (ii) outcrossing and selfing. The mating system is controlled by the **M** locus genotype of the mother, which is initially assumed to be fixed for allele *M*. With a mixture of polyandry and monandry, a fraction (Π) of gametes from *MM* females will be mated polyandrously (with many males competing for fertilisation) and the remaining fraction (1 − Π) is mated monandrously (with a single male). With a mixture of outcrossing and selfing, a fraction (Ω) of gametes from *MM* females are outcrossed and the remaining fraction (1 − Ω) are selfed. This way of modelling selfing has been called ‘prior selfing’ or ‘fixed selfing’, in contrast to mechanisms such as ‘delayed’, ‘mass action’, or ‘competing’ selfing [33, 41–44]. To examine mating system evolution, we introduce a new allele, *m*, that causes females to change their mating system allocation (to Π_*Mm*_ or Ω_*Mm*_ for *Mm* females and to Π_*mm*_ or Ω_*mm*_ for *mm* females). The spread of a modifier allele *m* that changes the mating system corresponds to mating system evolution.

Using male gametes for selfing may reduce the number of male gametes that are available for outcrossing; this is called ‘pollen discounting’ and has an important role in the evolution of selfing [45–47]. Although we follow convention by using the term ‘pollen discounting’, our models are also applicable to hermaphroditic animals (e.g., [48, 49]). In our model, pollen discounting is determined by *c*. When *c* = 0, selfing does not reduce the number of male gametes available for outcrossing. When *c* = 1, increased selfing results in a proportional decrease in the number of male gametes that are available for outcrossing. We assume that a consistent number of female gametes are fertilised per mother, which means we do not consider ‘seed discounting’ or ‘reproductive assurance’ [3, 43, 50].

We extrapolate the results from our two-locus model to multiple loci and then compare against explicit multi-locus simulations performed using SLiM v4.0.1 [51]. We simulated populations of 5,000 diploid hermaphrodites with genomes of 100 unlinked loci. Diploid parents are chosen as parents for the next generation according to their fitness across loci with fitness assumed to be multiplicative across loci. During gamete production, ten male gametes were produced for every female gamete. Female gametes that mate via polyandry or outcrossing sample male gametes produced by many males according to their gametic fitness. With selfing, a fraction 1 − Ω of female gametes were fertilised by sampling only from male gametes produced by the same individual. Monandry was implemented by designating a proportion 1 − Π of successful mothers as monandrous, whose full complement of female gametes would be fertilised by the male gametes of one other individual. When genomes comprised loci under and not under gametic selection, the loci were distributed randomly across the genome with a uniform distribution. To calculate inbreeding depression in a given generation, a second diploid population was populated by creating selfed offspring from individuals sampled to produce female gametes. Inbreeding depression in the previous generation is then calculated from the ratio of the mean fitness of the current generation to this selfed generation. Simulations were run for 50,000 generations, with statistics measured as an average over the final 5,000 generations over three replicates. These simulations were based on Recipes 16.15 and 16.16 in the SLiM handbook and the scripts are available in S3 File.

In summary, monandry and selfing both create a similar selective arena for haploid selection (equation S1–2 in S1 Appendix), in which gametes from only one male compete for fertilisation (Fig 1b). Polyandry and outcrossing create selective arenas where all male gametes in the population compete in a common pool (equation S1–3 in S1 Appendix). However, under monogamy, mating occurs between different individuals (equation S1–4 in S1 Appendix) whereas, under selfing, male gametes will fuse with female gametes produced by the same individual (equation S1–5 in S1 Appendix). Thus, selfing and monandry have similar direct effects on the intensity of haploid selection but selfing will also increase homozygosity.

## Results

We fist look at the allele frequency dynamics of a locus under selection with different mating systems. Initially, we assume all individuals have the same mating system by assuming they all carry the same modifier allele, *M*. We then allow the mating system to evolve by introducing a new modifier allele, *m*, that changes the mating system. To calculate these evolutionary trajectories, we assume that selection is weak (of order *ϵ* where *ϵ* ≪ 1). We further assume that the number of new mutations per locus per generation is very small (*μ* of order *ϵ*^3^). We extrapolate our results across multiple loci to approximate genome-wide mutation load and inbreeding depression. To do this, we assume that fitness effects across loci are uniform, multiplicative, and non-epistatic and that loci are loosely linked such that their frequencies can be considered independently (see S2 Appendix).

### Invasion and fixation

We first derive deterministic invasion conditions for two alleles at a single locus (**A**). If an allele’s frequency increases when it is rare, then it is able to invade. Invasion by allele *α* (either allele *A* or allele *a*, *α* ∈ {*A*, *a*}), is determined by *I*_*α*_. When *I*_*α*_ is positive (*I*_*α*_ > 0), selection favours the spread of a rare *α* allele (e.g., a new mutant). We express *I*_*α*_ by creating new compound parameters that describe the selection that occurs in females (${\bar{s}}_{\alpha }^{♀}$) and selection in males, which is further divided into diploid male selection (${\bar{s}}_{\alpha }^{♂,d}$) and male gametic selection (${\bar{s}}_{\alpha }^{♂,g}$) to give

$$\begin{array}{c} I_{\alpha }\approx \frac{{\bar{s}}_{\alpha }^{♀}}{2}+\frac{\left({\bar{s}}_{\alpha }^{♂,d}+{\bar{s}}_{\alpha }^{♂,g}\right)}{2}. \end{array}$$ (1)

For different mating systems (polyandry/monandry or outcrossing/selfing) we give these selection terms in Table 3. In short, *I*_*A*_ gives the selective advantage of a rare *A* allele among predominantly *a* alleles (vice versa for *I*_*a*_).

**Table 3:** Selection terms determining invasion under different mating systems.

stage	rare allele	term	polyandrous (Π) and/or monandrous (1 − Π)	outcrossing (Ω) and/or selfing (1 − Ω)
female	*A*	${\bar{s}}_{A}^{♀}$	$s_{Aa}^{♀}-s_{aa}^{♀}$	$\left(2-\Omega )(Fs_{AA}^{♀}+\left(1-F\right)s_{Aa}^{♀}-s_{aa}^{♀}\right)$
	*a*	${\bar{s}}_{a}^{♀}$	$s_{Aa}^{♀}-s_{AA}^{♀}$	$\left(2-\Omega )(Fs_{aa}^{♀}+\left(1-F\right)s_{Aa}^{♀}-s_{AA}^{♀}\right)$
male	*A*	${\bar{s}}_{A}^{♂,d}$	$s_{Aa}^{♂}-s_{aa}^{♂}$	$\Omega (Fs_{AA}^{♂}+\left(1-F\right)s_{Aa}^{♂}-s_{aa}^{♂})$
	*a*	${\bar{s}}_{a}^{♂,d}$	$s_{Aa}^{♂}-s_{AA}^{♂}$	$\Omega (Fs_{aa}^{♂}+\left(1-F\right)s_{Aa}^{♂}-s_{AA}^{♂})$
male gamete	*A*	${\bar{s}}_{A}^{♂,g}$	$s_{\Delta A}^{Aa}+\Pi ({\bar{s}}^{Aa}-s_{a}^{aa})$	$\Omega (s_{A}^{Aa}-s_{a}^{aa}+F(s_{A}^{AA}-s_{a}^{Aa}))$
	*a*	${\bar{s}}_{a}^{♂,g}$	$s_{\Delta a}^{Aa}+\Pi ({\bar{s}}^{Aa}-s_{A}^{AA})$	$\Omega (s_{a}^{Aa}-s_{A}^{AA}+F(s_{a}^{aa}-s_{A}^{Aa}))$

The selection terms in Table 3 highlight some important differences between mating systems. First, unlike monandry, selfing creates homozygotes such that homozygous fitnesses appear along with the inbreeding parameter (*F*), which indicates the excess of homozygotes relative to Hardy-Weinberg expectations and is *F* = (1 − Ω)/(1 + Ω) to leading order. Second, male and female fitnesses are weighted equally under monandry/polyandry but unequally when there is selfing. Increased selfing (lower Ω) effectively increases the importance of female fitness and decreases the importance of male fitness (as found without gametic competition [44]).

### Equilibrium allele frequency

We focus on two ways that genetic variation can be maintained over long time periods: mutation-selection balance and balancing selection. Under mutation-selection balance, mutational input maintains deleterious alleles (e.g., allele *a* when *I*_*a*_ < 0) despite selection removing them. Under balancing selection, both alleles are favoured when they are rare (*I*_*A*_ > 0 and *I*_*a*_ > 0) and allele frequency reaches a stable intermediate equilibrium with both alleles present.

#### Mutation-selection balance

Assuming that selection is weak and mutation rate is very small, the expected frequency of a deleterious allele at mutation-selection balance is

$$\begin{array}{c} {\hat{q}}_{\mu }\approx \frac{\mu }{-I_{a}}, \end{array}$$ (2)

which is the ratio of the rate at which new deleterious alleles arise by mutation and the rate they are removed by selection when rare. For a deleterious allele, *I*_*a*_ < 0 but the magnitude of *I*_*a*_ depends on the mating system and diploid and gametic selection according to (1) and Table 3 as described below.

**Fig 2:**
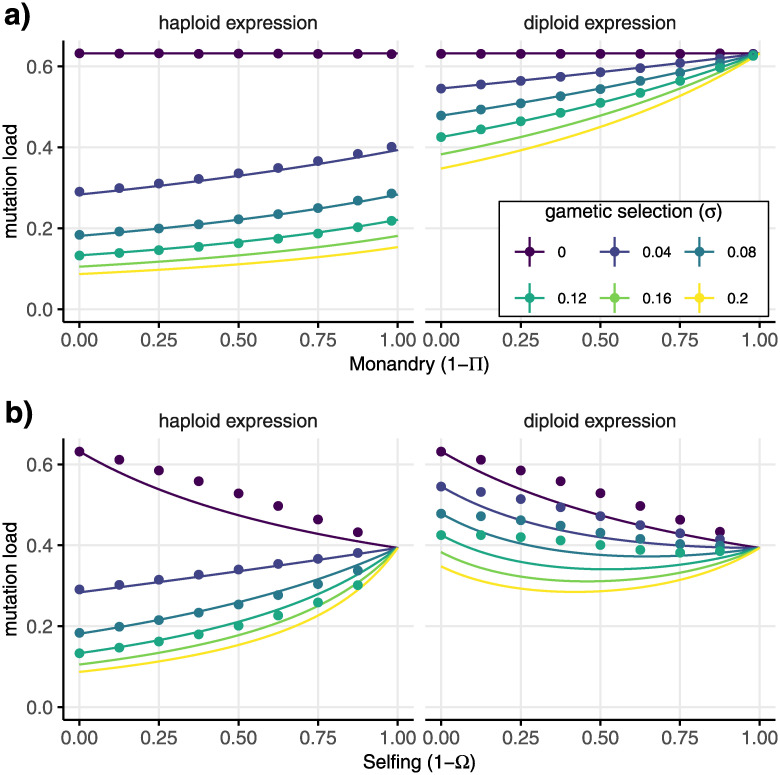
Mutation load for different mating systems and strengths of gametic selection. Mutation load is calculated for unlinked recessive deleterious alleles experiencing gametic selection ($s_{AA}^{♀}=s_{AA}^{♂}=0$, $s_{Aa}^{♀}=s_{Aa}^{♂}=-0.01$, $s_{aa}^{♀}=s_{aa}^{♂}=-0.05$, and *H* = 2), maintained by mutation at a rate of 1/2 per haploid genome per generation (*U* = *μl*_*μ*_ = 1/2). Lines show the approximated mutation load based on the equilibrium allele frequency in Eq (2) and the points show the outcome of multilocus simulations. The mating system varies (a) from polyandrous (Π = 1) to monandrous (Π = 0) or (b) from outcrossing (Ω = 1) to selfing (Ω = 0) and the colour shows the strength of gametic selection.

The maintenance of deleterious alleles through mutation decreases fitness, called ‘mutation load’. We extrapolate from the equilibrium allele frequency at a single locus (2) to approximate the mutation load across the genome, as described in S2 Appendix. This standard approximation method [27, 52, 53] assumes that the genotype frequencies of different loci can be considered independently and that fitness effects at different loci are multiplicative. We can evaluate the accuracy of this approximation against simulations (shown as points in Fig 2). As with previous results (e.g., Table 3 in [27], which does not include gametic selection), the approximation is least accurate for intermediate rates of selfing where associations between loci are likely to be most important. Nevertheless, the qualitative relationship between mutation load and expression pattern or mating system is consistent with expectations.

Monandry can only affect deleterious allele frequency if there is some gametic selection (see Table 3 and *σ* = 0 in Fig 2a). Gametic selection removes deleterious alleles and so reduces mutation load (*I*_*a*_ becomes more negative, see ${\bar{s}}_{a}^{♂,g}$ in Table 3 and Fig 2a). We find that monandry increases the frequency of deleterious mutations (increasing Π makes *I*_*a*_ more negative, see S1 File for proof). Monandry creates some highly competitive environments for gametes (from heterozygous males) and some highly uncompetitive environments (from homozygous males, see Fig 1b). However, the overall effect is that monandry reduces the intensity of gametic selection and increases mutation load under both haploid and diploid expression (Fig 2a). Haploid expression further decreases mutation load by directly exposing an allele’s deleterious effects to selection (compare panels in Fig 2a). That is, $s_{\Delta a}^{Aa}$ and ${\bar{s}}^{Aa}$ in Table 3 become more negative as diploid expression, *d*, decreases. Combining diploid gametic expression with monandry, on the other hand, prevents gametic selection altogether because there is no fitness variation among gametes produced by a single male with diploid expression (see Fig 1b for a diagrammatic representation and see the result in Fig 2a with diploid expression and monandry, Π = 0).

Fig 2b and Table 3 show a rather different relationship between selfing and mutation load. Selfing increases homozygosity, which means it exposes deleterious alleles to selection in homozygous adults, reducing mutation load even without gametic selection (i.e., Ω appears alongside diploid selection terms in Table 3), see *σ* = 0 in Fig 2b. Furthermore, because there are no heterozygotes in fully selfing populations, gametic selection has no effect on mutation load with either haploid or diploid expression (lines converge with Ω = 0 in Fig 2b and ${\bar{s}}_{a}^{♂,g}$ in Table 3 goes to zero). With outcrossing, gametic selection reduces mutation load, with larger reductions under haploid expression (compare panels in Fig 2b). Thus, selfing has opposing effects on mutation load: selfing reveals deleterious alleles to selection in homozygous adults and reduces mutation load but selfing reduces the efficiency of gametic selection at removing deleterious alleles. This means that selfing decreases mutation load without gametic selection but, when there is gametic selection, mutation load can increase with increased selfing because gametic selection becomes less effective (Fig 2b).

Overall, mutation load is predicted to be lower in genes that experience gametic selection, especially if they have haploid expression. The effect of gametic selection on mutation load is eliminated under selfing or under monandry with diploid expression. Generally, we predict the difference in mutation load between genes that are involved in gametic selection and those that are not becomes less as monandry and selfing becomes common. When comparing monandrous and polyandrous populations or species, we predict increased mutation load in the monandrous populations, all else being equal. Selfing can increase or decrease mutation load relative to outcrossing. Across the genome as a whole, selfing populations are likely to have lower mutation load, but we predict this effect is less strong (or even reversed) in the subset of genes that are involved in gametic selection.

#### Balancing selection

Balancing selection maintains genetic variation via selection. While it may be rare on a per-locus basis, balancing selection can account for an outsized fraction of genetic variation because the equilibrium allele frequencies can be high. A classic form of balancing selection is overdominance, where heterozygotes have a higher fitness than either of the two homozygotes. Other scenarios of balancing selection include sexually antagonistic selection, where one allele increases male fitness but decreases female fitness, and ploidally antagonistic selection, where one allele increases fitness during gametic selection but decreases fitness when expressed in the diploid adults. In all these forms of balancing selection, both alleles increase in frequency when rare (*I*_*A*_ > 0 and *I*_*a*_ > 0).

Under our weak selection assumptions, the equilibrium allele frequency of alleles maintained by balancing selection is

$$\begin{array}{c} {\hat{q}}_{B}\approx \frac{I_{a}}{I_{a}+I_{A}}, \end{array}$$ (3)

which reflects a balance between the advantage experienced by each allele when rare.

Both monandry and selfing decrease the overall intensity of gametic selection, despite creating some highly competitive environments (involving heterozygous males, Fig 1b). Thus, the allele favoured in male gametes is usually expected to be found at lower frequency with increasing monandry or selfing, although selfing also increases homozygosity and the balance of homozygous fitnesses can alter the allele frequency as well. Gamete-beneficial alleles are selected more strongly when they have haploid expression, which exposes alleles directly to selection, whereas diploid expression allows fitness effects to be masked. In S1 Fig, we show an example of balancing selection caused by ploidally antagonistic selection, where alleles have opposite fitness effects in gametes and adults, but these conclusions also apply to other forms of balancing selection where one allele has an advantage during gametic selection.

### Mating system evolution

We have shown that mating systems can determine how strongly alleles are favoured and shape the genetic variation that is maintained by mutations or balancing selection. Now, we explore how mating systems are expected to evolve when there is gametic selection. To examine the direction of mating system evolution, we evaluate the spread of a rare modifier allele (*m*) that changes the mating system. We assume that the modifier allele has no direct fitness effect. Therefore, there must be genetic variation at the selected **A** locus for mating system evolution to occur via its effects on gametic selection. We will assume that genetic variation is maintained by either mutation-selection balance or by balancing selection.

#### Evolution of polyandry versus monandry

First, we consider modifier alleles that increase or decrease the rate of polyandry versus monandry (indicated by superscript Π). When rare (e.g., a new mutant), the *m* allele frequency changes at rate λ^Π^ and will increase if λ^Π^ > 1. Assuming that the **A** locus is at mutation-selection balance (indicated by subscript *μ*), a rare modifier allele spreads at rate

$$\begin{array}{c} \lambda_{\mu }^{\Pi }\approx 1+\Delta_{\Pi }{\hat{q}}_{\mu }(s_{A}^{AA}-{\bar{s}}_{A}^{Aa})(-{\bar{w}}_{a}^{♀}-{\bar{w}}_{a}^{♂,d}+\Pi (s_{A}^{AA}-{\bar{s}}_{A}^{Aa})) \end{array}$$ (4)

where Δ_Π_ = (Π_*Mm*_ − Π)/2 is the increase in polyandry caused by the rare modifier allele. Because we assume that the *a* allele is deleterious at mutation-selection balance, all the other terms are positive (i.e., $s_{A}^{AA}-{\bar{s}}_{A}^{Aa}>0$, ${\bar{w}}_{a}^{♀}<0$ and ${\bar{s}}_{a}^{♂,d}$ when *a* is deleterious, see Table 3). Thus, modifier alleles will spread ($\lambda_{\mu }^{\Pi }>1$) if they increase the rate of polyandry (Δ_Π_ > 0). That is, we expect alleles at mutation-selection balance to favour the evolution of polyandry.

We also look at the evolution of polyandry versus monandry when genetic variation is maintained by balancing selection (indicated by subscript *B*). A mutant that alters the rate of polyandry will spread if $\lambda_{B}^{\Pi }>1$ where

$$\begin{array}{c} \lambda_{B}^{\Pi }\approx 1-\Delta_{\Pi }{\hat{q}}_{B}(1-{\hat{q}}_{B})s_{\Delta A}^{Aa}((1-{\hat{q}}_{B})(s_{A}^{AA}-{\bar{s}}^{Aa})+{\hat{q}}_{B}({\bar{s}}^{Aa}-s_{a}^{aa})) \end{array}$$ (5)

Unlike mutation-selection balance, balancing selection favours the evolution of monandry. That is, the modifier allele increases in frequency ($\lambda_{B}^{\Pi }>1$) when it increases monandry (Δ_Π_ < 0). The other factors in Eq (5), must combine to give a positive term as long as gametic fitness increases monotonically with increased expression of the higher fitness allele (e.g., when $s_{a}^{aa}\leq s_{a}^{Aa}<s_{A}^{Aa}\leq s_{A}^{AA}$, Fig 1a). However, when gametic expression is diploid, there is no fitness variation among gametes from male homozygotes ($s_{\Delta A}^{Aa}=0$) and no mating system evolution $\lambda_{B}^{\Pi }\approx 1$. Thus, as long as gametic expression is not diploid, loci under balancing selection will favour the evolution of monandry.

Post-copulatory sexual selection drives the evolution of polyandry or monandry in our model (Eqs 4 and 5) with mating systems evolving to increase offspring fitness. In the case of deleterious alleles maintained at mutation-selection balance, gametic selection increases offspring fitness by removing alleles that reduce fitness in both gametes and adults. Polyandry increases the efficacy of gametic selection (e.g., Fig 2). Therefore, offspring fitness is increased by evolving polyandry because it makes gametic selection more efficient.

Balancing selection on the other hand, favours the evolution of monandry. With balancing selection, gametic selection moves the equilibrium allele frequency away from the optimum for adults. This is clearly true when different alleles are favoured in gametes and adults (ploidally antagonistic selection), but also true for other forms of balancing selection, such as overdominance or sexually antagonistic selection. The result is that offspring fitness is increased by reducing the strength of gametic selection, which can be achieved by evolving monandry. Notably, mating system evolution is approximately neutral with diploid-like gametic expression because the gametic fitnesses become an extension of adult male fitness so gamete-beneficial alleles effectively benefit male offspring. Our results for mating system evolution are summarised in Table 4 and shown graphically in S2 Fig.

**Table 4:** Direction of mating system evolution.

Type of mating system variation	Genetic variation maintained by	Direction of mating system evolution
monandry/polyandry	mutation-selection balance	polyandry favoured
	balancing-selection	monandry favoured
	balancing-selection (diploid expression)	neutral
selfing/outcrossing*	mutation-selection balance (recessive allele)	outcrossing favoured
	mutation-selection balance (dominant allele)	selfing favoured
	balancing-selection	outcrossing favoured

* with full pollen discounting *c* = 1.

#### Evolution of outcrossing versus selfing

We next consider modifier alleles that increase or decrease the rate of outcrossing versus selfing (indicated by superscript Ω). To leading order, the evolution of outcrossing versus selfing is dominated by the direct transmission advantage of selfing. Specifically, to leading order, a rare modifier changes frequency at rate

$$\begin{array}{c} \lambda^{\Omega }\approx 1-\frac{\Delta_{\Omega }\left(1-c\right)}{2(1-c(1-\Omega ))} \end{array}$$ (6)

where Δ_Ω_ = (1 − *F*)(Ω_*Mm*_ − Ω) + *F*(Ω_*mm*_ − Ω) is the increase in outcrossing caused by the modifier. Without pollen discounting, selfers suffer no disadvantage in fertilising eggs from other individuals in outcrossing events. However, they monopolise the maternal and paternal contributions to zygotes formed from their own eggs, which gives selfing a strong intrinsic transmission advantage. To determine the (lower order) effect of gametic selection on the evolution of selfing, we now assume that there is complete pollen discounting (*c* = 1). That is, we assume that using male gametes for self fertilisation means proportionally fewer male gametes are available to outcross and fertilise others. This eliminates the transmission advantage of selfing (Eq 6) and allows us to examine the effects of selfing on offspring fitness. To simplify the results, we further assume that the modifier has a small and dominant effect on the rate of outcrossing (Δ_Ω_ is of order *ϵ* and Ω_*mm*_ = Ω_*Mm*_).

With these assumptions, a rare modifier that increases the rate of outcrossing by Δ_Ω_ will spread at rate

$$\begin{array}{c} \lambda_{\mu }^{\Omega }\approx 1+\Delta_{\Omega }(1+F){\hat{q}}_{\mu }\frac{\left(I_{A}+I_{a}\right)}{2\Omega } \end{array}$$ (7)

when deleterious alleles are maintained by mutation-selection balance (at frequency ${\hat{q}}_{\mu }$, Eq 2). Because the *a* allele is deleterious, *A* is favoured when rare (*I*_*A*_ > 0) and *a* is disfavoured when rare (*I*_*a*_ < 0), which means that selfing or outcrossing can evolve. However, most deleterious alleles are recessive [54–56], which means that the fitness difference between *AA* and *Aa* genotypes is less than the fitness difference between *Aa* and *aa* genotypes (specifically, $2s_{Aa}^{h}>s_{AA}^{h}+s_{aa}^{h}$ and $2{\bar{s}}^{Aa}>s_{A}^{AA}+s_{a}^{aa}$), giving *I*_*A*_+ *I*_*a*_ > 0. Thus, recessive deleterious alleles favour outcrossing ($\lambda_{\mu }^{\Omega }>1$ when Δ_Ω_ > 0).

When balancing selection maintains *a* alleles (at frequency ${\hat{q}}_{B}$, Eq 3), a mating system modifier spreads at rate

$$\begin{array}{c} \lambda_{B}^{\Omega }\approx 1+\Delta_{\Omega }(1+F){\hat{q}}_{B}(1-{\hat{q}}_{B})\frac{\left(I_{A}+I_{a}\right)}{2\Omega } \end{array}$$ (8)

which is similar to Eq (7) except $\hat{q}(1-\hat{q})$ is approximately $\hat{q}$ for rare alleles at mutation-selection balance. With balancing selection, both alleles have an advantage when they are rare (*I*_*A*_ > 0 and *I*_*a*_ > 0) and increased outcrossing is always favoured ($\lambda_{B}^{\Omega }>1$ when Δ_Ω_ > 0).

The summary in Table 4 contrasts selfing evolution with monandry evolution. Although selfing has the potential to affect offspring fitness via post-copulatory sexual selection, as with monandry, this is a lower order effect compared to increased homozygosity. That is, the modifier invasion fitnesses in Eqs (7) and (8) do not include post-copulatory sexual selection to leading order but instead include the relative fitness of heterozygotes versus homozygotes, *I*_*A*_ and *I*_*a*_. Balancing selection requires that homozygotes have a net disadvantage across the life cycle, so outcrossing is always favoured. This is clearly true when there is overdominance (e.g., [57–59]) but we show outcrossing continues to be favoured when there is also gametic selection, ploidally antagonistic and/or sexually antagonistic selection. Under mutation-selection balance, homozygotes also have a fitness disadvantage unless the deleterious alleles are dominant, which means that heterozygote fitness is lower than the average of the homozygotes. Thus, while monandry evolves via post-copulatory sexual selection in our model, selfing evolution is largely driven by its effects on homozygosity.

The conventional way to express the fitness consequences of increased homozygosity is ‘inbreeding depression’ (*δ*). Inbreeding depression is calculated as $\delta =1-{\bar{w}}_{s}/{\bar{w}}_{o}$ where ${\bar{w}}_{s}$ and ${\bar{w}}_{o}$ are the average fitnesses of offspring produced by selfing and outcrossing, respectively. Our results for selfing evolution can be re-stated in terms of inbreeding depression (see S2 Appendix for details). Specifically, if male gametic expression is haploid and there are no sex differences between sexes (i.e., $s_{A}^{Aa}=s_{A}^{AA}$, $s_{a}^{Aa}=s_{a}^{aa}$, $s_{g}^{♂}=s_{g}^{♀}$), Eqs (7) and (8) can be rewritten as $\lambda_{B}^{\Omega }\approx \lambda_{\mu }^{\Omega }\approx 1+\Delta_{\Omega }\left(1+F\right)\delta$. The (1 + *F*) term in Eqs (7) and (8) is because the *m* modifier allele is heterozygous with probability (1 − *F*), in which case it can pass on one copy of the *m* allele, and homozygous with probability *F*, in which case it will transmit two *m* alleles. Because we assume that the modifier has a dominant effect on the selfing rate, the increase/decrease in modifier frequency is the same for heterozygous or homozygous carriers and is given by their effect on the selfing rate, Δ_Ω_, multiplied by the inbreeding depression caused by the selected locus, *δ*.

In the two locus model, the strongest effect on selfing evolution comes from a transmission advantage (Eq 6), which can be as strong as 50%. Because selfing increases homozygosity, it can also create inbreeding depression, favouring outcrossing. A single locus can only create a small amount of inbreeding depression (*δ*), so this is a lower order effect in our two locus model (Eqs 7 and 8). However, the inbreeding depression produced by the combined effect of many selected loci ($\bar{\delta }$) could rival transmission advantage. Thus, we need to consider the genome wide inbreeding depression to determine whether selfing or outcrossing is favoured overall.

### Multiple loci

A single locus has a relatively weak effect on mating system evolution. In this section, we consider the net effect of many loci on mating system evolution. First, we approximate the total strength of selection for modifiers of monandry/polyandry. Second, we calculate inbreeding depression across many loci, which is a crucial determinant of selfing evolution.

To study many selected loci, we assume there is loose linkage and no epistasis so that we can ignore genetic associations between loci. This means the total indirect selection on a modifier of weak effect can be approximated by adding together the indirect selection caused by each locus, i.e., *s*_*tot*_ = ∑(λ_*l*_ − 1) where we sum over *l* loci to get the net selection on the mating system modifier (e.g., [60]). We consider three types of loci: there are *l*_*B*_ loci that experience balancing selection and *l*_*μ*_ loci with deleterious alleles at mutation-selection balance, of which a proportion *k* are expressed in gametes and (1 − *k*) only experience diploid selection (total *l* loci *l* = *l*_*B*_ + *l*_*μ*_). Within each of these categories, we assume that each locus is subject to the same selection coefficients for simplicity, but it is also possible to sum over a distribution of selective effects (e.g., [61–63]).

#### Net selection on polyandry versus monandry

**Fig 3:**
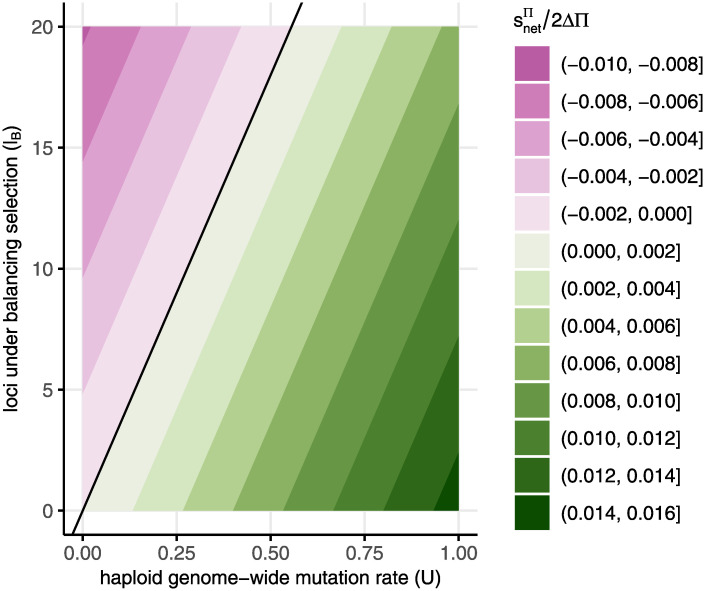
Net selection coefficient for polyandry ($s_{tot}^{\Pi }/\Delta_{\Pi }$) due to post-copulatory sexual selection. Green shows where increased polyandry is favoured and purple shows where monandry is favoured, with the magnitude of selection represented by contours. In this example, the population is initially monandrous (Π = 0) and $s_{tot}^{\Pi }/2\Delta_{\Pi }$ therefore corresponds to the rate of increase/decrease in frequency for a polyandry modifier (i.e., with Π_*Mm*_ = 1 and Δ_Π_ = 1/2). Because loci that do not experience gametic selection have no effect on the modifier, we only include loci that experience gametic selection (*k* = 1). Adult selection coefficients for deleterious mutations are the same as in Fig 2. Here, we assume balancing selection is ploidally antagonistic with allele *a* favoured during selection in adults ($s_{AA}^{♀}=s_{AA}^{♂}=-0.075$, $s_{Aa}^{♀}=s_{Aa}^{♂}=-0.01$, $s_{aa}^{♀}=s_{aa}^{♂}=0$) and allele *A* favoured in male gametes. Across all loci, the strength of gametic selection is *σ* = 0.12 and gametic expression is haploid-like (*d* = 0).

Fig 3 shows that a relatively small number of loci under balancing selection can have a disproportionately large impact on mating system evolution. Balancing selection maintains alleles at relatively high frequencies so that they can account for more genetic variation and thereby have stronger indirect effects on mating system modifiers. Because loci experiencing balancing selection favour monandry when they experience gametic selection, a small number of them could cancel out the selection for increased polyandry caused by a large number of loci at mutation-selection balance. Using our approximations, we do not need to specify the number of loci at mutation-selection balance but rather the genome-wide deleterious mutation rate, which is the product of the number of loci and the per locus mutation rate (*U* = *μl*_*μ*_).

The net selection coefficient for mating system modifiers can be used to evaluate the relative importance of gametic selection for mating system evolution. In Fig 3, the net strength of selection on modifiers of monandry varies up to 1.5%. Specifically, the strongest net selection on mating system for the parameters in Fig 3 would be for a polyandry modifier (2Δ_Π_ = 1) with a high rate of deleterious mutations *U* = 1 and no balancing selection (*l*_*B*_ = 0), giving *s*_*tot*_ = 0.015. This is the rate of increase in frequency of the rare polyandry allele, which is neutral other than changing post-copulatory sexual selection via the mating system. If we were to introduce a direct cost to the modifier of the same magnitude (e.g., reduces survival/fertility by 1.5%), then it would not spread.

#### Inbreeding depression and selfing evolution

The strongest evolutionary force in our two-locus model is the intrinsic transmission advantage of selfing but this can be rivalled by the inbreeding depression produced by many loci. We approximate the selection on selfing modifiers by summing the direct selection via transmission advantage (Eq 6) and the indirect selection across many loci (Eqs 7 and 8, which can be expressed using inbreeding depression as described above and in S2 Appendix). Focusing on an initially outcrossing population, this gives $s_{tot}=\bar{\delta }-\left(1-c\right)/2$, which is is equivalent to equation 15 in [64] where $\bar{\delta }$ is the total inbreeding depression across loci. That is, the transmission advantage of selfing is (1 − *c*)/2, reaching 50% when there is no pollen discounting (*c* = 0). This means that outcrossing is stable when inbreeding depression diminishes the fitness of selfed offspring by more than 50% ($\bar{\delta }>0.5$). The transmission advantage of selfing decreases with pollen discounting (higher *c*) such that outcrossing can be stable with less inbreeding depression.

**Fig 4:**
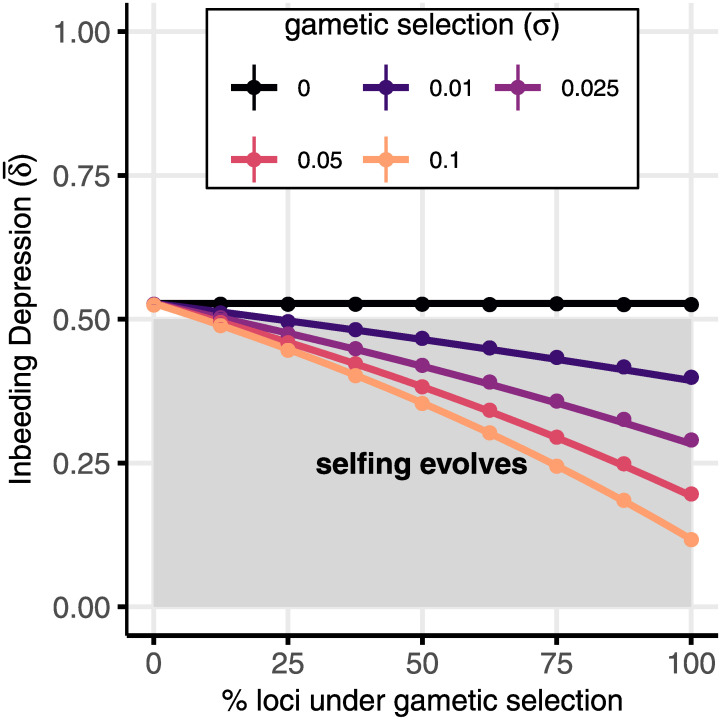
Gametic selection can significantly reduce inbreeding depression and thereby favour the evolution of selfing. Deleterious alleles have the same selection coefficients as in Fig 2. Inbreeding depression (y-axis) is caused by deleterious mutations, which occur at rate *U* = 1/2 across the genome, but only a fraction of loci experience gametic selection (x-axis). Here, gametic expression is haploid-like (*d* = 0) and there is no balancing selection *l*_*B*_ = 0, S3 Fig shows different levels of *U*, *d*, and *l*_*B*_. Inbreeding depression is calculated in an initially outcrossing population (Ω = 1) and our analytical approximation (lines) shows good agreement with the results of multilocus simulations (points). Outcrossing is stable to small-effect mating system modifiers unless inbreeding depression is below 1/2 (this threshold assumes no pollen discounting, *c* = 0), in which case selfing evolves (shaded area).

Fig 4 shows that gametic selection could have a large impact on inbreeding depression ($\bar{\delta }$, calculated from equation S2–5/S2–6), and therefore mating system evolution. For these parameters, reasonably weak gametic selection at a significant fraction of loci (e.g., *σ* = 0.01 at 50% of loci) decreases inbreeding depression enough to make outcrossing unstable when outcrossing would be stable without gametic selection (*σ* = 0, black line). Whether or not the presence of gametic selection determines the direction of mating system evolution depends on the balance between mutation rates and pollen discounting. With low mutation rates, inbreeding depression is also low and selfing will evolve unless pollen discounting is high. As pollen discounting increases, the transmission advantage of selfing decreases until it disappears (*c* = 1) and any inbreeding depression will favour outcrossing. That is, the position of the grey area in Fig 4 depends on pollen discounting and the position of the lines depends on the deleterious mutation rate (S3 Fig).

Inbreeding depression can be created by loci at mutation-selection balance and those experiencing balancing selection. Most of the genome carries deleterious mutations, with the relative importance of gametic selection depending on the fraction of these loci that experience gametic selection (*k*, x-axis in Fig 4). Gametic selection efficiently removes deleterious alleles, especially when expression is haploid (Fig 2 and S3 Fig), thereby reducing inbreeding depression and favouring selfing. As with monandry/polyandry evolution, a relatively small number of loci under balancing selection can have a disproportionately large effect on inbreeding depression because they can reach high frequencies (S3b Fig). As shown above, all forms of balancing selection increase inbreeding depression and favour outcrossing, including overdominance, sexually antagonistic selection, and ploidally antagonistic selection.

## Discussion

We found that monandry and selfing both decrease the efficacy of gametic selection but these mating systems follow different evolutionary trajectories. Monandry is favoured when alleles are maintained by balancing selection but not mutation-selection balance whereas selfing is not favoured in either scenario unless deleterious alleles are dominant (Table 4). The key difference between monandry and selfing is that selfing directly increases homozygosity. The increased homozygosity caused by selfing is more important than the effect of selfing on offspring fitness via post-copulatory sexual selection, which determines the evolution of polyandry in our model. Nevertheless, gametic selection has the potential to drastically reduce inbreeding depression by removing deleterious alleles (Fig 4), thereby causing selfing to evolve.

Despite creating some locally competitive environments (Fig 1b) selfing and monandry reduce responses to gametic selection. Responses to selection are also lessened with diploid expression due to masking effects. These patterns are in agreement with verbal arguments about the absence of pollen fitness variation under selfing [65] and models of sperm competition that do not include expression in other tissues [36, 37]. However, without expression in adults, genes expressed in male gametes have lessened evolutionary responses compared to genes expressed in both male and female adults for a given selection coefficient [66, 67]. Our models predict lower mutation load for genes that experience gametic selection, particularly those with haploid-expression or in polyandrous populations where gametic selection is more effective (Fig 2a). Unlike monandry, selfing is expected to decrease mutation load in the absence of gametic selection (Fig 2b). However, the fact that gametic selection becomes less effective means that selfing can increase mutation load for genes involved in gametic selection (Fig 2b). Overall, genes expressed in male gametes should have weaker signatures of mutation load than those that are not but the difference between these gene sets should be less with diploid gametic expression, monandry, and/or selfing.

Empirical results in plants demonstrate some of the expected differences in evolutionary rates for genes involved in gametic selection under outcrossing or selfing. Pollen-specific genes in outcrossing *Capsella grandiflora* had strong signatures of selection compared with seedling-specific genes [13], whereas no difference was found in a predominantly-selfing species, *Arabidopsis thaliana*, after accounting for tissue specificity [68], despite earlier reports, [69]. Across *Arabis alpina* populations, signatures of purifying selection on pollen-expressed genes are stronger where there is more outcrossing [70]. In both *C. grandiflora* and *A. alpina*, the signatures of selection are not elevated in genes specifically expressed in sperm cells, which are delivered to the female gametophyte by the pollen tissues. Thus, haploid expression alone cannot account for observed signatures of enhanced selection, which seem to also reflect selection on competitive ability during pollen germination and growth [70]. That is, while recessive lethal mutations should experience particularly strong selection in all haploid tissues, other alleles may experience lower selection coefficients in tissues that are not directly involved in gametic competition, such as plant sperm cells. Faster pollen tube growth rates are one indicator of male gametophyte competitive ability [11] that can evolve in response to the mating system [71]. For example, the predominantly outcrossing *Clarkia unguiculata* has faster pollen tube growth rates than in the closely related selfing species *C. exilis* [72]. One of our more counterintuitive results is that mutation load can increase, rather than decrease, for the subset of loci under strong gametic selection (Fig 2b, see also [31]), which could be tested using methods to estimate variant effects and mutation load from genomic data [73].

In animals, it is well established that a suite of sperm and seminal fluid proteins show elevated evolutionary rates (reviewed in [66, 74, 75] and a number of studies have examined these evolutionary rates across species with different mating systems (reviewed in [76]). For some genes, there is evidence that molecular evolution is correlated with polyandry [77–79], but this association is not ubiquitous and the predicted association may often be complicated by generation time, population size, and expression breadth [66, 67, 80, 81]. Single-cell transcriptomics now enable evolutionary rates to be compared between genes with expression patterns that vary from haploid-like to diploid-like in sperm [21]. Genes expressed during late stages of spermatogenesis seem to evolve particularly rapidly, which could reflect pleiotropic constraints and/or haploid expression [20]. It will be interesting to use analyses based on single-cell sperm transcriptomics to compare across populations or species with different mating systems, with stronger signatures of selection predicted for genes with more haploid-like expression and populations with more polyandry (see also [37]).

As well as evolutionary responses under different mating systems, we modelled the evolution of mating systems. Previous work found that females should evolve traits that increase the intensity of haploid gametic selection and thereby increase offspring fitness [30]. Our analysis of polyandry evolution shows that female mating rates can evolve to manipulate gametic selection. We find that increased offspring fitness can be achieved through increased polyandry as long as the direction of selection is the same in gametes and adults. This type of genetic variation is ephemeral or maintained by mutation. When genetic variation for gametic competitiveness is maintained by balancing selection, monandry is favoured because gametic selection moves allele frequencies away from their optimum.

We therefore show the source of correlations between gametic competitiveness and adult viability, which is crucial in previous models of polyandry evolution [38]. At equilibrium, we find that deleterious alleles at mutation-selection balance create the positive correlation that favours polyandry whereas balancing selection creates the opposite correlation and has an outsized effect because alleles can be maintained at high frequencies (Fig 3). Furthermore, we approximate the selective advantage of polyandry in terms of population genetic parameters such as the genome-wide deleterious mutation rate. For the parameters used in Fig 3, polyandry can only have a weak selective advantage of, at most, around 1.5%. A previous analysis concluded that the ‘sexy sperm’ effect is probably too weak to favour costly polyandry [82]. Our analysis also shows weak indirect benefits from ‘good sperm’ such that post-copulatory sexual selection may have a minor role in the evolution of polyandry, relative to other factors (reviewed in [34, 83–89]).

Our analysis of selfing evolution was focused on the impact of gametic selection and so we used approximations that neglect some well studied complications caused by associations between loci. First, viability loci can develop associations with each another and experience selective interference, which means extrapolating the inbreeding depression from the allele frequency at single loci becomes inaccurate [32, 61, 90–95]. For example, the shift from high to low inbreeding depression as selfing increases can happen more suddenly when accounting for associations between selected loci [32, 61]. Second, associations can build up between mating system modifiers of large effect and the background they create and these associations can favour selfing because selfing typically purges deleterious mutations [26, 52, 63, 90, 91, 96]. Gametic selection can lessen or reverse the tendency for selfing to purge deleterious mutations (Fig 2b), so we expect large effect selfing modifiers will generate comparatively less favourable genetic associations when there is gametic selection, but this remains to be investigated.

We found that increased offspring fitness through post-copulatory sexual selection had a weak effect on selfing evolution relative to the transmission advantage of selfing and increase in homozygosity. These are ‘genetic effects’ that neglect population dynamics and other ecological factors that might also influence selfing evolution [26]. For example, other models have incorporated sib-mating, population structure, or pollinator dynamics [42, 96–104]. Most importantly, our model excludes ‘reproductive assurance’ (selfing increases seed production), which is thought to be a major general driver of selfing evolution [3, 43, 50]. We suggest that the extent of haploid expression and the strength of gametic selection should be added to this list of factors governing variation in the prevalence of selfing between populations or taxonomic groups. We find that gametic selection can be a decisive determinant of mating system evolution, not through post-copulatory sexual selection, but by reducing inbreeding depression (Fig 4), especially with haploid expression.

Although they are inconspicuous life cycle stages, there is considerable potential for selection when pollen or sperm compete for fertilisation. The response to selection depends on the expression of genetic material in this competitive environment and the competitors involved, which is determined by the mating system. We have explored how organisms might evolve mating systems to optimise these evolutionary responses. Predicting variation in evolutionary responses under different mating systems and expression patterns offers a way to test our understanding of evolutionary processes more generally.

## Supporting information

S1 File Mathematica file. Contains recursion equations and derivations, can be used to replicate our results. Requires proprietary Mathematica software to open and run interactively. We provide S2 File, which can be used to view our analysis without proprietary software.
(NB)

S2 File Mathematica file (pdf version). A non-interactive version of S1 File containing our recursion equations, derivations, and analytical results.
(PDF)

S3 File SLiM scripts. Scripts to perform multilocus simulations using SLiM software v4.01.
(ZIP)

S1 Appendix Two-locus model details. Detailed description of the recursion equations describing genotype frequency changes.
(PDF)

S2 Appendix Mutation load and inbreeding depression. Extrapolates one locus equilibrium allele frequencies to get genome wide mutation load and inbreeding depression.
(PDF)

S1 FigEquilibrium allele frequency under balancing selection across mating systems.We plot the equilibrium frequency of the *A* allele ($1-{\hat{q}}_{B}$), which is favoured during gametic selection. Here, selection is ploidally antagonistic because allele *a* is favoured during selection in adults ($s_{AA}^{♀}=s_{AA}^{♂}=-0.05$ and $s_{aa}^{♀}=s_{aa}^{♂}=0$). Thus, no genetic variation is maintained without gametic selection (*σ* = 0). We assume beneficial effects are partially dominant ($s_{Aa}^{♀}=s_{Aa}^{♂}=-0.01$ and *H* = 2). The mating system varies (a) from polyandrous (Π = 1) to monandrous (Π = 0) or (b) from outcrossing (Ω = 1) to selfing (Ω = 0) and the colour shows the strength of gametic selection (*σ*).(EPS)

S2 Fig Selection for rare alleles that increase the rate of (a) monandry or (b) selfing. Negative values indicate selection for increased (a) polyandry or (b) outcrossing. That is, increased outcrossing is favoured when loci are at mutation-selection balance or under balancing selection but increased polyandry is only favoured when the selected locus is at mutation-selection balance. Colours indicate to degree to which expression in male gametes is haploid or diploid. With balancing selection and diploid expression (yellow), there is no selection on the modifier of polyandry. The *A* allele is assumed to be favoured in male gametes (*σ* = 0.12). Alleles at mutation-selection balance are partially recessive (as in Fig 2) and balancing selection is ploidally antagonistic (as in S1 Fig). We assume complete pollen discounting (*c* = 1) such that selfing does not have a direct transmission advantage.
(EPS)

S3 FigThe relationship between gametic selection and inbreeding depression with (a) haploid or diploid expression and different genome-wide deleterious mutation rates and (b) balancing selection.Gametic selection reduces inbreeding depression, but diploid expression (dashed lines in a) weakens this effect. Whether gametic selection determines the direction of mating system evolution depends on the genome-wide deleterious mutation rate (*U*, across panels in a) and pollen discounting. Without pollen discounting (*c* = 0), outcrossing is stable to small-effect mating system modifiers unless inbreeding depression is below 1/2, in which case selfing evolves (light grey area). The threshold level of inbreeding depression, above which outcrossing is stable, reduces linearly with increased pollen discounting (e.g., dark grey area shows threshold for *c* = 1/2). Adult selection coefficients for deleterious mutations are the same as in Figs 2 and 4. In (b), loci under balancing selection increase inbreeding depression (dashed lines in b). Here, balancing selection results from overdominance in adults ($s_{AA}^{♀}=s_{AA}^{♂}=-0.075$, $s_{Aa}^{♀}=s_{Aa}^{♂}=0$, $s_{aa}^{♀}=s_{aa}^{♂}=-0.05$) and gametic selection (*σ* = 0.025).(EPS)
