# Supplementary material for: Gametic selection favours polyandry and selfing
Source: PLoS Genet. 2024 Feb 16;20(2):e1010660. doi: 10.1371/journal.pgen.1010660 (PMC10903963; doi:10.1371/journal.pgen.1010660)
Supplement: S2 File — A non-interactive version of S1 File containing our recursion equations, derivations, and analytical results. (PDF) [file pgen.1010660.s002.pdf]

Gametic selection favours polyandry and selfing

Michael Francis Scott, Carl Mackintosh, and Simone Immler

Introduction

We assume two loci: a selected locus (A) with two alleles *A*/*a*, and a modifier (M) with alleles *M*/*m* that determines the mating system. When considering the evolution of polyandry/monandry, the modifier determines the fraction of a female's offspring that will be produced through monandry or through polyandry. When considering the evolution of selfing, the modifier determines the fraction of a female's offspring that will be produced via selfing or via outcrossing. We also use these two locus results to approximate the effect of many selected loci.

We use the following fitness notation in the Mathematica notebook. When selection is assumed to be weak, we take a Taylor series in the small parameter ϵ, in which case we convert to the δW notation.

Fitness in male gametes

A-bearing from AA father	WAhom	= 1+δWAhom*ϵ
a-bearing from AA father	WAhom	= 1+δWahomAA*ϵ
A-bearing from Aa father	WAhom	= 1+δWAhet*ϵ
a-bearing from Aa father	WAhom	= 1+δWahet*ϵ
a-bearing from aa father	Wahom	= 1+δWahom*ϵ

Fitness in diploids

AA	WAAk	= 1+δWAAk
Aa	WAak	= 1+δWAak
aa	Waak	= 1+δWaak

where k=m for males and k=f for females (or for the fitness effect on male/female function in hermaphrodites), $k \in \{m,f\}$.

Diploid frequencies:

In this model, we census the frequency of diploid zygotes, given by x[i,j], where i and j give the haplotypes carried ($i \in \{1,2,3,4\}$, $j \in \{1,2,3,4\}$) indicating the following haplotypes:
subscript haplotype

1	MA
2	Ma
3	mA
4	ma

Monandry/Polyandry
In this model, the parameter of interest is Πij, which determines the fraction of offspring produced via polyandry (Πz) versus monandry (1-Πz), where z is the diploid genotype at the M locus in the mother (z ∈ {MM, Mm, mm}).
Selfing/Outcrossing
In this model, the parameter of interest is Ωij, which determines the fraction of offspring produced via outcrossing (Ωz) versus selfing (1-Ωz), where z is the diploid genotype at the M locus in the mother (z ∈ {MM, Mm, mm}).

(0) Shared functions [enter]

Functions [enter]

We census immediately after zygote formation, where the two sexes are equal in frequency. Because it doesn’t matter to the next generation whether a haplotype was inherited from the mother or father (no parental effects), we can reduce the number of genotypes by counting together the genotypes together involving {i,j} and {j,i}, getting the 10 unique genotypes:
{MA/MA, MA/Ma, MA/mA, MA/ma, Ma/Ma, Ma/mA, Ma/ma, mA/mA, mA/ma, ma/ma}
The indices {1,2,3,4} correspond to haplotypes {MA, Ma, mA, ma}, so the diploid genotypes are given by the indices in “genotypes”

```
In[•]:= genotypes = Flatten[Table[{i, j}, {i, 1, 4}, {j, i, 4}], 1]
Out[•]= {{1, 1}, {1, 2}, {1, 3}, {1, 4}, {2, 2}, {2, 3}, {2, 4}, {3, 3}, {3, 4}, {4, 4}}
```

The following functions give an index for the A or M genotype based on the haplotype index: 1=A and 2=a and 1=M and 2=m. getHap returns the haplotype from the A and B genotypes.

```
In[•]:= Ageno[k_] := Mod[Ceiling[k / 1], 2, 1]
Mgeno[k_] := Mod[Ceiling[k / 2], 2, 1]
getHap[a_, b_] := a + 2 b - 2
```

Selection in the diploid phase.
In the section on monandry/polyandry, we assume separate males and females. In the section on selfing, we are interested in hermaphrodites, where viability selection will affect both sexual functions equally. However, even in hermaphrodites we allow for separate male and female fitnesses in case of sexually antagonistic fertility selection. Therefore, we allow separate male and female fitnesses.
The following gives fitness vectors to be applied to the genotype frequencies.

```
In[ ]:= femfit = WAAf * Boole[Map[Ageno[#[[1]]] * Ageno[#[[2]]] == 1 &, genotypes]] +
          WAaf * Boole[Map[(Ageno[#[[1]]] * Ageno[#[[2]]] == 2) &, genotypes]] +
          Waaf * Boole[Map[Ageno[#[[1]]] * Ageno[#[[2]]] == 4 &, genotypes]];
        malefit = WAAm * Boole[Map[Ageno[#[[1]]] * Ageno[#[[2]]] == 1 &, genotypes]] +
          WAam * Boole[Map[(Ageno[#[[1]]] * Ageno[#[[2]]] == 2) &, genotypes]] +
          Waam * Boole[Map[Ageno[#[[1]]] * Ageno[#[[2]]] == 4 &, genotypes]];
```

Use these fitness vectors to get the mean fitness of males and females and the frequencies of the different genotypes after selection (femfreq and malefreq) Neither locus is linked to the sex determining region (or individuals are hermaphrodite) so the genotype frequencies among males and females are the same before selection (given by x[{i,j}]).

```
In[ ]:= meanfemfit = Total[Map[x, genotypes] * femfit];
        meanmalefit = Total[Map[x, genotypes] * malefit];
        femfreq = Map[x, genotypes] * femfit / meanfemfit;
        malefreq = Map[x, genotypes] * malefit / meanmalefit;
```

The normalisation step (mean fitness) means that these are frequencies among males and females and will sum to 1:

```
In[ ]:= Simplify[Total[femfreq]]
        Simplify[Total[malefreq]]

Out[ ]= 1

Out[ ]= 1
```

We assume that mutation occurs from A->a at rate μ. These give indicators of which gametes are A-bearing or a-bearing based on the genotype vectors.

```
In[ ]:= Agenotypes = Boole[Map[Ageno[#] == 1 &, Table[x, {x, 1, 4}]]];
        agenotypes = Boole[Map[Ageno[#] == 2 &, Table[x, {x, 1, 4}]]];
```

The transmission function captures recombination and mutation

In[]:=
```
Clear[transmission]
transmission[{x_, y_}] :=
 Block[{}, (* this function will return a vector of the length 4,
  giving the genotypes of gamete/gametophytes
   produced by mother/father with genotype x y *)
  type = {0, 0, 0, 0};
  (*before accounting for recombination,
  the gametes to carry the parental haplotypes*)
  type⟦x⟧ = type⟦x⟧ + 1 / 2;
  type⟦y⟧ = type⟦y⟧ + 1 / 2;

  (*the x haplotype can exchange a single allele at the A or M locus with the
   y haplotype, these return the genotype code for each of these exchanges*)
  switchA = getHap[Ageno[y], Mgeno[x]];
  switchM = getHap[Ageno[x], Mgeno[y]];

  type⟦x⟧ = type⟦x⟧ - (r / 2);
  (*recombinants remove the original haplotype combinations*)
  type⟦switchA⟧ = type⟦switchA⟧ + (r / 2); (*add recombinant types*)
  type⟦y⟧ = type⟦y⟧ - r / 2;
  (*recombinants remove the original haplotype combinations*)
  type⟦switchM⟧ = type⟦switchM⟧ + r / 2; (*add recombinant types*)

  (*modify genotype frequencies by mutation*)
  Simplify[(1 - μ) * Agenotypes * type + (*remove A genotypes that mutate*)
    agenotypes * type + (*a genotypes unchanged*)
    RotateRight[(μ) * Agenotypes * type]
   (*add A genotypes that mutate to a genotypes*)]
  (*n.b. this assumes that the haplotypes are shifted by one via the
    RotateRight function so can only be used when mutations go from A→
   a and these indices are successively coded*)
 ]
```

For polyandry and outcrossing, we need to get the common gamete pool, summed over all father genotype frequencies (xm[{i,j}]) and the gametes they each produce and the respective fitnesses of each of those gamete types:

In[]:=
```
Clear[gametefit]
gametefit[{x_, y_}] :=
 Block[{}, (* this function will return a vector of the fitness
   of gametes produced by father with genotype x y *)
  Which[
   Ageno[x] * Ageno[y] == 1, {WAhom, WahomAA, WAhom, WahomAA},
   Ageno[x] * Ageno[y] == 2, {WAhet, Wahet, WAhet, Wahet}, (* fitness of
    the four types of gametes/gametophytes produced by heterozygotes
     (allows A-bearing and a-bearing to have different fitnesses) *)
   Ageno[x] * Ageno[y] == 4, {Wahom, Wahom, Wahom, Wahom}]
 ]
```

Gamete Fitness Function [enter]

Gametic fitness can take up to four values depending on the genotype of the gamete and the genotype of the father.

Numerically, we will assume that gametic fitness is a function of the amount of expression of the A versus a alleles. Thus, the gametic fitness of A-bearing and a-bearing gametes produced by Aa heterozygotes depends on the degree of transcript sharing among gametes.

We assume that A-bearing gametes from AA homozygotes have fitness 1 and a-bearing gametes from aa homozygotes have fitness 1-t. For gametes from Aa heterozygotes, we use the parameter d for the degree of diploid-like expression (transcript sharing between gametes gives diploid-like expression). A-bearing gametes from Aa heterozygotes have fitness 1-t*γ[d/2,H] and a-bearing gametes from Aa heterozygotes have fitness 1-t*γ[1-d/2,H]. The function γ[x,H] defines a continuous form of allelic dominance. We will assume that $\gamma[x] = \left(1 - \left(1 - (x)^{H}\right)^{1/H}\right)$ where x is the % of a allele transcripts and H defines the shape of the relationship between transcript percentage and fitness. When H>1, sharing transcripts increases the average fitness of haploids produced by heterozygotes (A allele partially dominant). When H<1, the A allele is partially recessive.

In[ ]:=
```
Clear[tryγ]
tryγ[x_, H_] = (1 - (1 - (x)^H)^(1/H));
Show[
 Plot[Evaluate@(1 - t * tryγ[x, H] /. t → 0.1 /. H → {1 / 2, 1, 2}), {x, 0, 1},
  AspectRatio → 1, AxesLabel → {"% a expression", "Hap Fitness"},
  PlotStyle → {{Red}, {Black}, {Blue}}],
 Graphics[{Text["H=2", {0.5, 0.993}],
   Text["H=1", {0.5, 0.955}], Text["H=1/2", {0.5, 0.913}]}]
]
Show[Plot[
  {1, 1 - t * tryγ[d/2, 2] /. t → 0.1, 1 - t * tryγ[1 - d/2, 2] /. t → 0.1, 1 - t /. t → 0.1},
  {d, 0, 1}, AspectRatio → 1, AxesLabel → {"allele sharing (d)", "Hap fitness"},
  PlotStyle → {{Orange}, {Purple}, {Purple}, {Green}},
  PlotRange → {1 - 0.1 - 0.005, 1.005 + 0.005}],
 Graphics[{Text["A-bearing from AA", {0.5, 1.005}],
   Text["A-bearing from Aa", {0.5, 0.99}], Text["a-bearing from Aa",
    {0.5, 0.952}], Text["a-bearing from aa", {0.5, 0.905}]}]
]
```

Out[ ]=

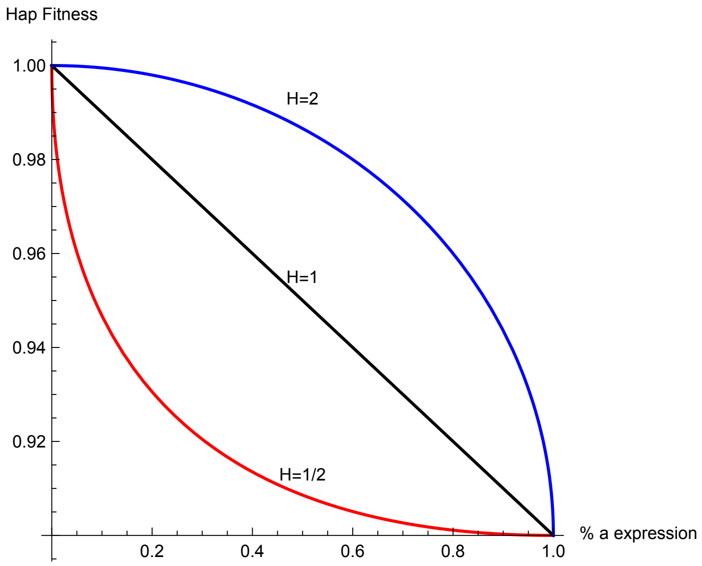

Out[ ]=

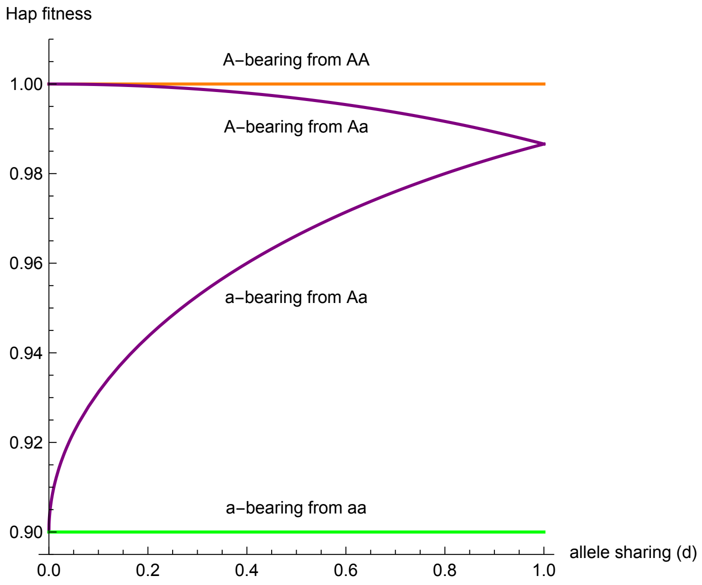

Replacements [enter]

Some useful replacements for assuming weak selection and/or migration and/or modifier effects.

In[ ]:=
```
fitnessDiffs = {
   WAA → 1 + δWAA,
   WAa → 1 + δWAa,
   Waa → 1 + δWaa,
   WAAf → 1 + δWAAf,
   WAaf → 1 + δWAaf,
   Waaf → 1 + δWaaf,
   WAAm → 1 + δWAAm,
   WAam → 1 + δWAam,
   Waam → 1 + δWaam,
   WAhom → 1 + δWAhom,
   WahomAA → 1 + δWahomAA,
   WAhet → 1 + δWAhet,
   Wahet → 1 + δWahet,
   Wahom → 1 + δWahom
  };
```

In[ ]:=
```
weaksel = {
   δWAA → δWAA * ϵ,
   δWAa → δWAa * ϵ,
   δWaa → δWaa * ϵ,
   δWAAf → δWAAf * ϵ,
   δWAaf → δWAaf * ϵ,
   δWaaf → δWaaf * ϵ,
   δWAAm → δWAAm * ϵ,
   δWAam → δWAam * ϵ,
   δWaam → δWaam * ϵ,
   δWAhom → δWAhom * ϵ,
   δWahomAA → δWahomAA * ϵ,
   δWAhet → δWAhet * ϵ,
   δWahet → δWahet * ϵ,
   δWahom → δWahom * ϵ
  };
```

In[ ]:=
```
weakΩmod = {Ωmm → ΩMM + δΩmm * ϵ, ΩMm → ΩMM + δΩMm * ϵ};
```

```
In[ ]:= noSexDiff = {
    WAAf → WAA,
    WAaf → WAa,
    Waaf → Waa,
    WAAm → WAA,
    WAam → WAa,
    Waam → Waa,
    δWAAf → δWAA,
    δWAaf → δWAa,
    δWaaf → δWaa,
    δWAAm → δWAA,
    δWAam → δWAa,
    δWaam → δWaa
   };
```

(1) Gametic Selection and Monandry/Polyandry

Recursions

Recursions

Function to get the fraction of gametes from diploid parent with genotype xy that will be mated with one mating partner

```
In[ ]:= Clear[monandry]
monandry[{x_, y_}] := Block[{},
   Which[
    Mgeno[x] * Mgeno[y] == 1, 1 - πMM,
    Mgeno[x] * Mgeno[y] == 2, 1 - πMm,
    Mgeno[x] * Mgeno[y] == 4, 1 - πmm]
   (* depends on the diploid M locus genotype*)
  ]
```

Get numbers of each gamete genotype after selection in polyandous pool.

In[•]:=
```
gametePoolNum = Total[(*sum over the gamete genotypes from different males*)
     malefreq * (* diploid male genotype frequencies after selection *)
      Flatten[ (*Flatten table to level 1 to get list of 10 ij genotypes,
       matching the malefreqs *)
       Table[(* table over male i and j haplotypes *)
        transmission[{i, j}] (* gamete types produced *) *
         gametefit [{i, j}](* fitness of those gamete types *)
        , {i, 1, 4}
        , {j, i, 4}], 1]] // FullSimplify;
```

Convert to gamete frequencies

In[•]:=
```
gameteMeanFit = Total[gametePoolNum] // Simplify;
gametePoolFreq = gametePoolNum / gameteMeanFit // Simplify;
```

In[•]:=
```
Simplify[Total[gametePoolFreq]] (* check that frequencies sum to 1 *)
```

Out[•]=
```
1
```

Next, we calculate the offspring produced from all the different combinations of males and females.

In[ ]:=
```
kids = Table[0, {i, 1, 4}, {j, i, 4}];
kidsFromMono = Table[0, {i, 1, 4}, {j, i, 4}];
(* initialise tables in which to put kids haplotype frequencies *)
kidsFromPoly = Table[0, {i, 1, 4}, {j, i, 4}];
For[n = 1, n ≤ Length[genotypes], n++,
 (*Summing over all mothers, i.e., all 16 combinations of
  i and j haplotypes given by the genotypes vector above *)
 mum = genotypes⟦n⟧;
 eggsforMono = monandry[mum] * transmission[mum];
 (*Calculating female gametes created,
 including mutation and recombination, destined for monoandry*)
 eggsforPoly = (1 - monandry[mum]) * transmission[mum];
 (*Calculating female gametes created,
 including mutation and recombination, destined for polyandry*)
 For[m = 1, m ≤ Length[genotypes], m++, (*Summing over all fathers, i.e., all
   combinations of i and j haplotypes given by the genotypes vector above *)
  dad = genotypes⟦m⟧;
  malegametes = transmission[dad];
  (*Calculating male gametes received, including mutation and recombination*)
  afterGameticSelectionMono =
   (malegametes * gametefit[dad]) / Total[malegametes * gametefit[dad]];
  (*Male gamete pool after selection*)
  mapPatHaplotypesMono = malefreq⟦m⟧ * femfreq⟦n⟧ *
    Transpose[{eggsforMono}].{afterGameticSelectionMono};
  kidsFromMono =
   kidsFromMono + Simplify[Table[If[i == j, mapPatHaplotypesMono⟦i, j⟧,
       (mapPatHaplotypesMono⟦i, j⟧ + mapPatHaplotypesMono⟦j, i⟧)],
      {i, 1, 4}, {j, i, 4}]] (*combine the kids produced i eggs
    and j pollen and those from j eggs and i pollen if i=/=j *)
 ];
 mapPatHaplotypesPoly = femfreq⟦n⟧ * Transpose[{eggsforPoly}].{gametePoolFreq} ;
 (* the contributions from this mother via
  polygamous mating are determined by the total pollen pool *)
 kidsFromPoly = kidsFromPoly + Simplify[
    Table[If[i == j, mapPatHaplotypesPoly⟦i, j⟧, (mapPatHaplotypesPoly⟦i, j⟧ +
        mapPatHaplotypesPoly⟦j, i⟧)], {i, 1, 4}, {j, i, 4}] ]
    (*combine the kids produced i eggs and j pollen and those
   from j eggs and i pollen if i=/=j *)
]
kids = kids + kidsFromMono + kidsFromPoly;
```

These sum to one:

In[ ]:=
```
newkid = Flatten[kids, 1];
Simplify[Total[newkid]]
```

Out[ ]=
```
1
```

Equilibrium (M fixed)

Calculating the Equilibrium (M fixed)

With M fixed, we seek to determine q and F, the equilibrium frequencies of a and the departure from Hardy-Weinberg among the zygotes (before diploid/sporophytic selection).

Here, we pick the recursions where M is fixed and re-write using q and F:

```
In[∘]:= Mfixed = Map[Mgeno[#⟦1⟧] == 1 && Mgeno[#⟦2⟧] == 1 &, genotypes];
recursionsMfixed = Pick[newkid, Mfixed];
genotypesMfixed = Map[x, Pick[genotypes, Mfixed]];
qFreplace = Thread[genotypesMfixed →
    {(1 - q)^2 * (1 - F) + F * (1 - q), 2 q (1 - q) * (1 - F), q^2 * (1 - F) + F * q}];
```

Here, we set the other genotype frequencies (involving the m allele) to 0 and convert the recursions to difference equations. Therefore, roots of “solveme” are equilibria.

```
In[∘]:= solveme =
  Factor[(recursionsMfixed - genotypesMfixed) /. qFreplace /. x[{i_, j_}] → 0];
```

Balancing Selection (equilibrium maintained by selection)

Balancing Selection Equilibrium

We input the weak selection assumptions and neglect mutation to calculate the equilibrium (qsol)

```
In[∘]:= Fsol0 = Flatten[
  Solve[Normal[Series[(solveme /. fitnessDiffs /. weaksel /. μ → 0), {ϵ, 0, 0}]] ==
    {0, 0, 0}, F]]
Out[∘]= {F → 0}
```

```
In[∘]:= qsol = Solve[Normal[
     Series[(solveme /. fitnessDiffs /. weaksel /. μ → 0 /. F → (F /. Fsol0) + δF * ϵ),
      {ϵ, 0, 1}]] == {0, 0, 0}, {q, δF}] // Simplify
```

Solve : Equations may not give solutions for all "solve" variables.

```
Out[∘]= {{q → 0}, {q → 1},
  {q → -((2 δWAaf - 2 δWAAf + 2 δWAam - 2 δWAAm + δWahet - δWAhet + δWahet ΠMM + δWAhet ΠMM -
        2 δWAhom ΠMM) / (2 (δWaaf - 2 δWAaf + δWAAf + δWaam - 2 δWAam + δWAAm -
         δWahet ΠMM - δWAhet ΠMM + δWahom ΠMM + δWAhom ΠMM))), δF → 0}}
```

Internal Stability Conditions

First, we get the jacobian matrix when M is fixed. The solution for the characteristic polynomial at equilibria q=0 and q=1 give the eigenvalues that determine instability of these equilibria.

```
In[ ]:= jacobMfixed =
          Transpose[Map[D[recursionsMfixed, #] &, genotypesMfixed]] /. qFreplace /.
           x[{i_, j_}] → 0;

In[ ]:= charpoly0 = Det[(jacobMfixed /. q → 0 /. F → 0 /. μ → 0 // Simplify) -
            IdentityMatrix[Length[genotypesMfixed]] * λ] // Simplify;
        charpoly1 = Det[(jacobMfixed /. q → 1 /. F → 0 /. μ → 0 // Simplify) -
            IdentityMatrix[Length[genotypesMfixed]] * λ] // Simplify;
```

We can get the stability conditions assuming weak selection using the weaksel substitution.

```
In[ ]:= δλ1sol0 =
         δλ1 /. Solve[Normal[Series[charpoly0 /. fitnessDiffs /. weaksel /. λ → 1 + δλ1 * ϵ,
              {ϵ, 0, 1}]] == 0, δλ1][[1]]
        δλ1sol1 =
         δλ1 /. Solve[Normal[Series[charpoly1 /. fitnessDiffs /. weaksel /. λ → 1 + δλ1 * ϵ,
              {ϵ, 0, 1}]] == 0, δλ1][[1]]
```

Out[ ]= $\frac{1}{4}$ (2 δWAaf − 2 δWAAf + 2 δWAam − 2 δWAAm + δWahet − δWAhet + δWahet ΠMM + δWAhet ΠMM − 2 δWAhom ΠMM)

Out[ ]= $\frac{1}{4}$ (−2 δWaaf + 2 δWAaf − 2 δWaam + 2 δWAam − δWahet + δWAhet + δWahet ΠMM + δWAhet ΠMM − 2 δWahom ΠMM)

These can be written in the following form:

In[ ]:= δλ1sol0 − $\left(\frac{\text{δWAaf − δWAAf + δWAam − δWAAm}}{2} + \frac{\text{δahetdiff + ΠMM (δhetmean − δWAhom)}}{2}\right)$ /.
δahetdiff → $\frac{\text{δWahet − δWAhet}}{2}$ /. δhetmean → $\frac{\text{δWahet + δWAhet}}{2}$ // Simplify

δλ1sol1 − $\left(\frac{\text{δWAaf − δWaaf + δWAam − δWaam}}{2} + \frac{\text{−δahetdiff + ΠMM (δhetmean − δWahom)}}{2}\right)$ /.
δahetdiff → $\frac{\text{δWahet − δWAhet}}{2}$ /. δhetmean → $\frac{\text{δWahet + δWAhet}}{2}$ // Simplify

Out[ ]= 0

Out[ ]= 0

We can also see that the equilibria can be written in terms of these instability conditions.

In[ ]:= Ia = δλ1sol0;
IA = δλ1sol1;
q /. qsol[[3]] // Factor;
$\frac{\text{Ia}}{\text{IA + Ia}}$ // Simplify;
%% / % // Factor

Out[ ]= 1

Frequency of allele favoured during gametic selection increases with

polyandry

The change in allele frequency with increasing polyandry can be seen from the partial derivative of q w.r.t. polyandry rate, which we re-write as $\frac{-2q\,((1-q)\,(\delta WAhom-\delta hetmean)+q\,(\delta hetmean-\delta Wahom))}{4\,\lambda 0}$

In[•]:=
```
Simplify[D[q /. qsol[[3]], ΠMM]];
Simplify[% /. Solve[{δλ1sol0 == λ0, δλ1sol1 == λ1}, {δWAaf, δWAAm}]];
Flatten[Simplify[% /. Solve[λ0/(λ0 + λ1) == q, λ1]]];
(-2 q ((1 - q) (δWAhom - δhetmean) + q (δhetmean - δWahom)))/(4 λ0) - % /.
  δhetmean → (δWahet + δWAhet)/2 // Simplify
```

Out[•]= {0}

This is negative assuming that the a allele is less fit during gametic selection. δhetmean is the average fitness of gametes from heterozygous males ($\frac{(\delta Wahet+\delta WAhet)}{2}$). When the a allele is monotonically deleterious δhetmean<δWAhom and δhetmean>δWahom, the whole term is negative.

Deleterious Mutations (equilibrium maintained at mutation-selection balance)

Mutation-Selection Balance Equilibrium

Here, we will assume weak selection and very weak mutation.

In[•]:=
```
Normal[Series[(solveme /. fitnessDiffs /. weaksel /. μ → μ * ϵ^3), {ϵ, 0, 0}]];
Flatten[Solve[% == 0, F]]
```

Out[•]= {F → 0}

Next, we find the solution for balancing selection again.

In[•]:=
```
Normal[Series[
   (solveme /. fitnessDiffs /. weaksel /. μ → μ * ϵ^3 /. F → δF * ϵ), {ϵ, 0, 1}]];
Solve[% == 0, {q, δF}]
```

Solve : Equations may not give solutions for all "solve" variables.

Out[•]=
```
{{q → 0}, {q → 1},
 {q → (-2 δWAaf + 2 δWAAf - 2 δWAam + 2 δWAAm - δWahet + δWAhet - δWahet ΠMM -
      δWAhet ΠMM + 2 δWAhom ΠMM) / (2 (δWaaf - 2 δWAaf + δWAAf + δWaam - 2 δWAam +
        δWAAm - δWahet ΠMM - δWAhet ΠMM + δWahom ΠMM + δWAhom ΠMM)), δF → 0}}
```

For mutation selection-balance, we are interested in the lower order terms for the equilibrium around q=0.

In[ ]:=
```
Normal[
  Series[solveme /. fitnessDiffs /. weaksel /. μ → μ * ϵ^3 /. F → δF * ϵ /. q → q1 * ϵ,
   {ϵ, 0, 2}]];
Flatten[Solve[% == 0, {q1, δF}]]
```

Solve : Equations may not give solutions for all "solve" variables.

Out[ ]= {q1 → 0}

Taking the next order in ϵ

In[ ]:=
```
Normal[
  Series[solveme /. fitnessDiffs /. weaksel /. μ → μ * ϵ^3 /. F → δF * ϵ /. q → q2 * ϵ^2,
   {ϵ, 0, 3}]];
qsolMS = Simplify[Flatten[Solve[% == {0, 0, 0}, {q2, δF}]] /. ϵ → 1]
```

Out[ ]= {q2 → - ((4 μ) / (2 δWAaf - 2 δWAAf + 2 δWAam - 2 δWAAm + δWahet - δWAhet + δWahet ΠMM + δWAhet ΠMM - 2 δWAhom ΠMM)), δF → 0}

The invasion terms from the balancing selection section above give the fitness of allele a when rare, which is the denominator of this equilibrium:

In[ ]:=
$$Ia = \left(\frac{\delta WAaf - \delta WAAf + \delta WAam - \delta WAAm}{2} + \frac{\delta ahetdiff + \Pi MM\ (\delta hetmean - \delta WAhom)}{2}\right) /. \ \delta ahetdiff \to \frac{\delta Wahet - \delta WAhet}{2} /. \ \delta hetmean \to \frac{\delta Wahet + \delta WAhet}{2} \ // \ Simplify;$$
$$\frac{\mu}{-Ia} - q2 \ /. \ qsolMS \ // \ Simplify$$

Out[ ]= 0

Frequency of allele favoured during gametic selection increases with polyandry

The change in allele frequency with increasing polyandry can be seen from the partial derivative of q w.r.t. polyandry rate, which we re-write as
$$\frac{-\mu\ (\delta WAhom - \delta hetmean)}{2\ Ia^2}$$

In[ ]:=
```
D[(q2 /. qsolMS), ΠMM] // Simplify;
```
$$\frac{-\mu\ (\delta WAhom - \delta hetmean)}{2\ Ia^2} - \% \ /. \ Ia \to \left(\frac{\delta WAaf - \delta WAAf + \delta WAam - \delta WAAm}{2} + \frac{\delta ahetdiff + \Pi MM\ (\delta hetmean - \delta WAhom)}{2}\right) /. \ \delta ahetdiff \to \frac{\delta Wahet - \delta WAhet}{2} /. \ \delta hetmean \to \frac{\delta Wahet + \delta WAhet}{2} \ // \ Simplify$$

Out[ ]= 0

The denominator is squared and therefore positive. The numerator is negative when we assume that a is deleterious so the frequency of the deleterious allele decreases with polyandry.

Mutation Load

Here, we are going to assume that fitness effects of multiple loci are multiplicative.
Following Charlesworth & Charlesworth (1992), fitness with j homozygotes and i heterozygotes is
$w_{ij} = (1 - s)^j (1 - h\, s)^i$.
Or
$w_{ij} = (\text{Waa})^j (\text{WAa})^i (\text{WAA})^{1-i-j}$ in our notation.
Assuming we have l loci that are unlinked, the expected values for j and i are just the expected genotype frequencies based on the allele frequency. We assume a fraction k of loci experience selection in male gametes and the other loci experience only diploid selection.

```
In[•]:= freqaa1 = (q1^2 (1 - F) + q1 F);
freqAa1 = (2 q1 (1 - q1) (1 - F));
freqAA1 = ((1 - q1)^2 (1 - F) + F (1 - q1));
freqaa2 = (q2^2 (1 - F) + q2 F);
freqAa2 = (2 q2 (1 - q2) (1 - F));
freqAA2 = ((1 - q2)^2 (1 - F) + F (1 - q2));
meanFitMS = Normal[Series[(Waa)^(l (k freqaa1+(1-k) freqaa2))
        (WAa)^(l (k freqAa1 + (1-k) freqAa2)) (WAA)^(l (k freqAA1 + (1-k) freqAA2)) /. q1 → (μ * ϵμ)/(-δλ1 * ϵ) /.
      q2 → ((μ * ϵμ)/(-δλ1nohapsel * ϵ)) /. noSexDiff /. fitnessDiffs /. weaksel,
    {ϵ, 0, 2}, {ϵμ, 0, 1}]] /. ϵ → 1 /. ϵμ → 1 // Simplify

Out[•]= ⅇ^((l (2 (δWAa-δWAA)+F (δWaa-2 δWAa+δWAA)) ((-1+k) δλ1-k δλ1nohapsel) μ)/(δλ1 δλ1nohapsel))
```

This is written in a simplified form in the appendix

```
In[•]:= fitTerm = ((2 - F) ( δWAA) - 2 (1 - F) (δWAa ) - F (δWaa ));
Exp[-l fitTerm ( k q + (1 - k) q2 ) ] - meanFitMS /. δλ1 → -μ / q /.
  δλ1nohapsel → -μ / q2 // Simplify

Out[•]= 0
```

Evolution of polyandry

General

We now calculate the external stability of the M-fixed equilibria by looking at the recursion equations involving the m allele and assuming that it is rare.

```
In[•]:= malleles = Map[(Mgeno[#[[1]]] == 2 || Mgeno[#[[2]]] == 2) &, genotypes];
recursionsExt = Pick[newkid, malleles];
genotypesExt = Map[x, Pick[genotypes, malleles]];
differenceExt = recursionsExt - genotypesExt;
jacobExtFull =
  Factor[Transpose[Map[D[recursionsExt, #] &, genotypesExt]] /. qFreplace /.
    x[{i_, j_}] → 0];
```

This is a block triangular matrix, where the last three rows are immaterial (consisting of mm

homozygotes):

```
In[ ]:= jacobExtFull[[5 ;; Dimensions[jacobExtFull][[1]],
          1 ;; Dimensions[jacobExtFull][[2]]]] // Factor // MatrixForm
```

Out[ ]//MatrixForm=

$$\begin{pmatrix} 0 & 0 & 0 & 0 & 0 & 0 & 0 \\ 0 & 0 & 0 & 0 & 0 & 0 & 0 \\ 0 & 0 & 0 & 0 & 0 & 0 & 0 \end{pmatrix}$$

We can then drop the last three rows and columns, to get a new 4x4 matrix:

```
In[ ]:= jacobExt = jacobExtFull[[1 ;; 4, 1 ;; 4]] // Factor;
```

The leading eigenvalue determining external instability can be found by solving the following characteristic polynomial for λ:

```
In[ ]:= charpoly = Det[IdentityMatrix[4] * λ - jacobExt];
```

If the m allele increases in frequency when rare (λ>1), then it invades and the polyandry rate evolves.

Modifier evolution with balancing selection

We approximate λ using our weak selection assumptions and look at sequential terms in a Taylor Series approximation. The logic of this analysis can be found in Otto & Day (2008) Chapter 12. To leading order, λ=1.

```
In[ ]:= Normal[Series[charpoly /. fitnessDiffs /. weaksel /. μ → 0, {ϵ, 0, 0}]];
        Solve[% == 0, λ] // Flatten

Out[ ]= {λ → 0, λ → 0, λ → 1, λ → 1 - r}
```

Here, we input the equilibrium solution from qsol. We have tried including terms in q to order ϵ and terms in F to order ϵ^2 to confirm that these don't affect λ.

```
In[ ]:= Normal[Series[(charpoly /. λ → 1 + δλ1 * ϵ /. fitnessDiffs /. weaksel /. μ → 0 /.
              q → (q /. qsol[[3]]) /. F → 0), {ϵ, 0, 1}]];
        δλ1solExt = Flatten[Factor[Solve[% == 0, δλ1]]]

Out[ ]= {δλ1 → 0}
```

```
In[ ]:= Normal[Series[(charpoly /. λ → 1 + δλ2 * ϵ^2 /. fitnessDiffs /. weaksel /. μ → 0 /.
              q → (q /. qsol[[3]]) /. F → 0), {ϵ, 0, 2}]];
        δλ2solExt = Flatten[Factor[Solve[% == 0, δλ2]]]
```

$$\Big\{\delta\lambda 2 \to -\Big(\Big((\delta Wahet - \delta WAhet)\ \big(\delta Waaf\ \delta Wahet - \delta WAAf\ \delta Wahet + \delta Waam\ \delta Wahet - \delta WAAm\ \delta Wahet + \delta Wahet^2 + \delta Waaf\ \delta WAhet - \delta WAAf\ \delta WAhet + \delta Waam\ \delta WAhet - \delta WAAm\ \delta WAhet - \delta WAhet^2 - 2\ \delta WAaf\ \delta Wahom + 2\ \delta WAAf\ \delta Wahom - 2\ \delta WAam\ \delta Wahom + 2\ \delta WAAm\ \delta Wahom - \delta Wahet\ \delta Wahom + \delta WAhet\ \delta Wahom - 2\ \delta Waaf\ \delta WAhom + 2\ \delta WAaf\ \delta WAhom - 2\ \delta Waam\ \delta WAhom + 2\ \delta WAam\ \delta WAhom - \delta Wahet\ \delta WAhom + \delta WAhet\ \delta WAhom\big)\ (-\Pi Mm + \Pi MM)\ (-2\ \delta Waaf + 2\ \delta WAaf - 2\ \delta Waam + 2\ \delta WAam - \delta Wahet + \delta WAhet + \delta Wahet\ \Pi MM + \delta WAhet\ \Pi MM - 2\ \delta Wahom\ \Pi MM)\ (2\ \delta WAaf - 2\ \delta WAAf + 2\ \delta WAam - 2\ \delta WAAm + \delta Wahet - \delta WAhet + \delta Wahet\ \Pi MM + \delta WAhet\ \Pi MM - 2\ \delta WAhom\ \Pi MM)\Big) \Big/ \Big(32\ (-\delta Waaf + 2\ \delta WAaf - \delta WAAf - \delta Waam + 2\ \delta WAam - \delta WAAm + \delta Wahet\ \Pi MM + \delta WAhet\ \Pi MM - \delta Wahom\ \Pi MM - \delta WAhom\ \Pi MM)^3\Big)\Big)\Big\}$$

Re-written as

```
In[]:= (δλ2 /. δλ2solExt) /.
    Solve[{(q /. {q → -((2 δWAaf - 2 δWAAf + 2 δWAam - 2 δWAAm + δWahet - δWAhet +
                    δWahet ΠMM + δWAhet ΠMM - 2 δWAhom ΠMM) / (2 (δWaaf - 2 δWAaf + δWAAf +
                       δWaam - 2 δWAam + δWAAm - δWahet ΠMM - δWAhet ΠMM + δWahom ΠMM +
                       δWAhom ΠMM))), δF → 0}) == q}, {δWAAm}][[1]] // Simplify;
  -q (1 - q) ((ΠMm - ΠMM)/2) ((δWAhet - δWahet)/2) ((1 - q) (δWAhom - (δWahet + δWAhet)/2) +
       q ((δWahet + δWAhet)/2 - δWahom)) - % // Simplify

Out[]= 0
```

Modifier evolution with mutation selection balance

As above, we approximate λ using our weak selection and weak mutation assumptions and look at sequential terms in a Taylor Series approximation. The logic of this analysis can be found in Otto & Day (2008) Chapter 12. To leading order, λ=1.

```
In[]:= Normal[Series[
    charpoly /. μ → ϵ^3 μ /. fitnessDiffs /. weaksel /. F → δF2 * ϵ^2, {ϵ, 0, 0}]];
  Solve[% == 0, λ] // Flatten

Out[]= {λ → 0, λ → 0, λ → 1, λ → 1 - r}

In[]:= Normal[
   Series[(charpoly /. λ → 1 + δλ1 * ϵ /. μ → ϵ^3 μ /. fitnessDiffs /. weaksel /. F → 0),
    {ϵ, 0, 1}]];
  δλ1solExtMS = Flatten[Factor[Solve[% == 0, δλ1]]]

Out[]= {δλ1 → 0}

In[]:= Normal[Series[
    (charpoly /. λ → 1 + δλ2 * ϵ^2 /. μ → ϵ^3 μ /. fitnessDiffs /. weaksel /. F → 0) /.
      q → q2 * ϵ^2 /. qsolMS, {ϵ, 0, 2}]];
  δλ2solExtMS = Flatten[Factor[Solve[% == 0, δλ2]]]

Out[]= {δλ2 → 0}

In[]:= Normal[Series[
    (charpoly /. λ → 1 + δλ3 * ϵ^3 /. μ → ϵ^3 μ /. fitnessDiffs /. weaksel /. F → 0) /.
      q → q2 * ϵ^2 /. qsolMS, {ϵ, 0, 3}]];
  δλ3solExt = Flatten[Factor[Solve[% == 0, δλ3]]]

Out[]= {δλ3 → 0}
```

In[ ]:=
```
Normal[Series[
    (charpoly /. λ → 1 + δλ4 * ϵ^4 /. μ → ϵ^3 μ /. fitnessDiffs /. weaksel) /. F → 0 /.
     q → (q2 * ϵ^2 /. qsolMS), {ϵ, 0, 4}]];
δλ4solExt = Flatten[Factor[Solve[% == 0, δλ4]]]
```

Out[ ]=
```
{δλ4 → ((δWahet + δWAhet - 2 δWAhom) μ (-ΠMm + ΠMM)
    (2 δWAaf - 2 δWAAf + 2 δWAam - 2 δWAAm + δWahet ΠMM + δWAhet ΠMM - 2 δWAhom ΠMM)) /
  (2 (2 δWAaf - 2 δWAAf + 2 δWAam - 2 δWAAm + δWahet - δWAhet +
      δWahet ΠMM + δWAhet ΠMM - 2 δWAhom ΠMM))}
```

which can be re-written

In[ ]:=
```
q ((δλ4 /. δλ4solExt /. F → 0) / (q2 /. qsolMS)) // Simplify;
q (ΠMm - ΠMM)/2 (δWAhom - (δWahet + δWAhet)/2) ( ((δWAAm - δWAam) + (δWAAf - δWAaf)) +
    ΠMM (δWAhom - (δWahet + δWAhet)/2)); // Simplify
% - %% // Simplify
```

Out[ ]=
```
0
```

Because we assume that there is mutation from A->a and that the a allele is maintained at mutation-selection balance, the a allele must be selected against.
The sign of this term is therefore the same as the sign of (ΠMm-ΠMM), which is positive for any modifier that increases the rate of polyandry. Increased polyandry is therefore favoured.

(2) Gametic Selection and and Selfing/Outcrossing

Recursions

Recursions

Function to get the fraction of female gametes from a parent with genotype xy that will be mated through selfing

In[ ]:=
```
Clear[selfing]
selfing[{x_, y_}] := Block[{},
   Which[
    Mgeno[x] * Mgeno[y] == 1, 1 - ΩMM,
    Mgeno[x] * Mgeno[y] == 2, 1 - ΩMm,
    Mgeno[x] * Mgeno[y] == 4, 1 - Ωmm]
   (* depends on the diploid M locus genotype*)
  ]
```

Function to apply pollen discounting to the male gametes exported by a male with genotype xy.

```
In[ ]:= Clear[pollenDiscounting]
pollenDiscounting[{x_, y_}] := Block[{},
  Which[
   Mgeno[x] * Mgeno[y] == 1, 1 - (1 - ΩMM) * c,
   Mgeno[x] * Mgeno[y] == 2, 1 - (1 - ΩMm) * c,
   Mgeno[x] * Mgeno[y] == 4, 1 - (1 - Ωmm) * c]
  (* depends on the diploid M locus genotype*)
 ]
```

Get numbers of each male gamete genotype after selection in outcrossing pool.

```
In[ ]:= gametePoolNum = Total[(*sum over the gamete types from different males*)
     malefreq * (* diploid male genotype frequencies after selection *)
      Flatten[ (*Flatten table to level 1 to get list of 10 ij genotypes,
       matching the malefreqs *)
       Table[(* table over male i and j haplotypes *)
        transmission[{i, j}] (* male gamete types produced *) *
         pollenDiscounting[{i, j}] *
         (*all outcross male gametes may be discounted by a factor
          that depends on the rate of selfing in the adult parent*)
         gametefit [{i, j}](* fitness of those male gamete types *)
        , {i, 1, 4}
        , {j, i, 4}], 1]] // FullSimplify;
```

Convert to male gamete frequencies

```
In[ ]:= gameteMeanFit = Total[gametePoolNum] // Simplify;
gametePoolFreq = gametePoolNum / gameteMeanFit // Simplify;
```

```
In[ ]:= Simplify[Total[gametePoolFreq]] (* check that frequencies sum to 1 *)
Out[ ]= 1
```

```
In[•]:= kids = Table[0, {i, 1, 4}, {j, i, 4}];
(* initialise tables in which to put kids haplotype frequencies *)
kidsfromSelf = Table[0, {i, 1, 4}, {j, i, 4}];
kidsfromOut = Table[0, {i, 1, 4}, {j, i, 4}];
For[n = 1, n ≤ Length[genotypes], n++,
 (*Summing over all mothers, i.e., all combinations of i
  and j haplotypes given by the genotypes vector above *)
 self = genotypes⟦n⟧;
 eggsforSelf = (selfing[self]) transmission[self];
 (*Calculating female gametes created,
 including mutation and recombination, destined for selfing*)
 gametesSelf = transmission[self];
 (*Calculating male gametes received from self,
 including mutation and recombination*)
 afterGameticSelectionSelf =
  (gametesSelf * gametefit[self]) / Total[gametesSelf * gametefit[self]];
 (*Self male gamete frequencies after selection*)
 mapPatHaplotypesSelf =
  femfreq⟦n⟧ * Transpose[{eggsforSelf}].{afterGameticSelectionSelf};
 kidsfromSelf = kidsfromSelf + Simplify[
    Table[If[i == j, mapPatHaplotypesSelf⟦i, j⟧, (mapPatHaplotypesSelf⟦i, j⟧ +
         mapPatHaplotypesSelf⟦j, i⟧)], {i, 1, 4}, {j, i, 4}]] ;
 (*combine the kids produced i female gametes and j male gametes
   and those from j female gametes and i male gametes if i=/=j *)

 eggsforOut = (1 - selfing[self]) transmission[self];
 (*Calculating female gametes created,
 including mutation and recombination, destined for outcrossing*)
 mapPatHaplotypesOut = femfreq⟦n⟧ * Transpose[{eggsforOut}].{gametePoolFreq};
 kidsfromOut = kidsfromOut + Simplify[
    Table[If[i == j, mapPatHaplotypesOut⟦i, j⟧, (mapPatHaplotypesOut⟦i, j⟧ +
         mapPatHaplotypesOut⟦j, i⟧)], {i, 1, 4}, {j, i, 4}] ]
    (*combine the kids produced i female gametes and j male gametes
   and those from j female gametes and i male gametes if i=/=j *)
]
kids = kids + kidsfromSelf + kidsfromOut;
```

These sum to one:

```
In[•]:= newkid = Flatten[kids, 1];
Simplify[Total[newkid]]

Out[•]= 1
```

Equilibrium (M fixed)

Calculating the Equilibrium (M fixed)

With M fixed, we seek to determine q and F, the equilibrium frequencies of a and the departure from Hardy-Weinberg among the zygotes (before diploid/sporophytic selection).

Here, we pick the recursions where M is fixed and re-write using q and F:

In[∘]:=
```
Mfixed = Map[Mgeno[#[[1]]] == 1 && Mgeno[#[[2]]] == 1 &, genotypes];
recursionsMfixed = Pick[newkid, Mfixed];
genotypesMfixed = Map[x, Pick[genotypes, Mfixed]];
qFreplace = Thread[genotypesMfixed →
    {(1 - q)^2 * (1 - F) + F * (1 - q), 2 q (1 - q) * (1 - F), q^2 * (1 - F) + F * q}];
```

Here, we set the other genotype frequencies (involving the m allele) to 0 and convert the recursions to difference equations. Therefore, roots of “solveme” are equilibria.

In[∘]:=
```
solveme =
  Factor[(recursionsMfixed - genotypesMfixed) /. qFreplace /. x[{i_, j_}] → 0];
```

Balancing Selection (equilibrium maintained by selection)

Calculating the equilibrium (M fixed) - weak selection

We input the weak selection assumptions and neglect mutation to calculate the equilibrium (qsol)

In[∘]:=
```
Fsol0 = Flatten[
  Solve[Normal[Series[(solveme /. fitnessDiffs /. weaksel /. μ → 0), {ϵ, 0, 0}]] ==
    {0, 0, 0}, F]]
```

Out[∘]= $\left\{F \to -\frac{-1+\Omega MM}{1+\Omega MM}\right\}$

Thus, the inbreeding coefficient is $\frac{(1-\Omega MM)}{1+\Omega MM}$ to leading order. i.e., if there is any selfing (ΩMM<1) then there will be an excess of homozygotes (positive F). In calculating the equilibrium allele frequency q, we also get deviations in F of order ϵ.

In[∘]:=
```
qsol = Solve[Normal[
     Series[(solveme /. fitnessDiffs /. weaksel /. μ → 0 /. F → (F /. Fsol0) + δF * ϵ),
      {ϵ, 0, 1}]] == {0, 0, 0}, {q, δF}] // Simplify
```

Solve : Equations may not give solutions for all "solve" variables.

Out[∘]= $\{\{q \to 0\}, \{q \to 1\},$
$\{q \to (-\Omega MM\ (\delta WAAm - \delta Wahet + \delta WAhet - \delta Wahom + \delta WAhom + 2\ \delta WAaf\ (-2 + \Omega MM) +$
$\delta Waam\ (-1 + \Omega MM) - 2\ \delta WAam\ \Omega MM + \delta WAAm\ \Omega MM - \delta Wahet\ \Omega MM - \delta WAhet\ \Omega MM +$
$\delta Wahom\ \Omega MM + \delta WAhom\ \Omega MM) + \delta Waaf\ (2 - 3\ \Omega MM + \Omega MM^2) + \delta WAAf\ (-2 - \Omega MM + \Omega MM^2)) /$
$(2\ \Omega MM\ (-2\ \delta WAAf + \delta Waaf\ (-2 + \Omega MM) - 2\ \delta WAaf\ (-2 + \Omega MM) + \delta WAAf\ \Omega MM - \delta Waam\ \Omega MM +$
$2\ \delta WAam\ \Omega MM - \delta WAAm\ \Omega MM + \delta Wahet\ \Omega MM + \delta WAhet\ \Omega MM - \delta Wahom\ \Omega MM - \delta WAhom\ \Omega MM)),$
$\delta F \to ((-1 + \Omega MM)\ (-8\ \delta WAAf^2\ \delta Waam + 8\ \delta WAAf^2\ \delta WAam + 4\ \delta WAAf^2\ \delta Wahet + 4\ \delta WAAf^2\ \delta WAhet -$
$8\ \delta WAAf^2\ \delta Wahom + 4\ \delta WAAf\ \delta Waam^2\ \Omega MM - 4\ \delta WAAf\ \delta Waam\ \delta WAam\ \Omega MM -$
$4\ \delta WAAf\ \delta Waam\ \delta WAAm\ \Omega MM + 4\ \delta WAAf\ \delta WAam\ \delta WAAm\ \Omega MM + 4\ \delta WAAf\ \delta Waam\ \delta Wahet\ \Omega MM -$
$8\ \delta WAAf\ \delta WAam\ \delta Wahet\ \Omega MM + 4\ \delta WAAf\ \delta WAAm\ \delta Wahet\ \Omega MM - 4\ \delta WAAf\ \delta Wahet^2\ \Omega MM -$
$8\ \delta WAAf\ \delta Waam\ \delta WAhet\ \Omega MM + 8\ \delta WAAf\ \delta WAam\ \delta WAhet\ \Omega MM + 4\ \delta WAAf\ \delta WAhet^2\ \Omega MM +$

$8 \delta WAAf \delta Waam \delta Wahom \Omega MM - 4 \delta WAAf \delta WAam \delta Wahom \Omega MM - 4 \delta WAAf \delta WAAm \delta Wahom \Omega MM +$
$4 \delta WAAf \delta Wahet \delta Wahom \Omega MM - 8 \delta WAAf \delta WAhet \delta Wahom \Omega MM +$
$4 \delta WAAf \delta Wahom^2 \Omega MM - 4 \delta WAAf \delta Waam \delta WAhom \Omega MM + 4 \delta WAAf \delta WAam \delta WAhom \Omega MM +$
$4 \delta WAAf \delta Wahet \delta WAhom \Omega MM - 4 \delta WAAf \delta Wahom \delta WAhom \Omega MM +$
$8 \delta WAaf^3 (-2 + \Omega MM)^2 \Omega MM - 2 \delta WAAf^2 \delta Waam \Omega MM^2 + 2 \delta WAAf \delta Waam^2 \Omega MM^2 +$
$2 \delta WAAf^2 \delta WAam \Omega MM^2 - 2 \delta WAAf \delta Waam \delta WAam \Omega MM^2 - 2 \delta WAAf \delta Waam \delta WAAm \Omega MM^2 +$
$2 \delta WAAf \delta WAam \delta WAAm \Omega MM^2 - 3 \delta WAAf^2 \delta Wahet \Omega MM^2 + 2 \delta WAAf \delta Waam \delta Wahet \Omega MM^2 -$
$\delta Waam^2 \delta Wahet \Omega MM^2 - 4 \delta WAAf \delta WAam \delta Wahet \Omega MM^2 + 2 \delta Waam \delta WAam \delta Wahet \Omega MM^2 +$
$2 \delta WAAf \delta WAAm \delta Wahet \Omega MM^2 - 2 \delta WAam \delta WAAm \delta Wahet \Omega MM^2 + \delta WAAm^2 \delta Wahet \Omega MM^2 -$
$2 \delta WAAf \delta Wahet^2 \Omega MM^2 + 2 \delta WAam \delta Wahet^2 \Omega MM^2 - 2 \delta WAAm \delta Wahet^2 \Omega MM^2 +$
$\delta Wahet^3 \Omega MM^2 + 5 \delta WAAf^2 \delta WAhet \Omega MM^2 - 4 \delta WAAf \delta Waam \delta WAhet \Omega MM^2 +$
$\delta Waam^2 \delta WAhet \Omega MM^2 + 4 \delta WAAf \delta WAam \delta WAhet \Omega MM^2 - 2 \delta Waam \delta WAam \delta WAhet \Omega MM^2 +$
$2 \delta WAam \delta WAAm \delta WAhet \Omega MM^2 - \delta WAAm^2 \delta WAhet \Omega MM^2 + 2 \delta Waam \delta Wahet \delta WAhet \Omega MM^2 -$
$4 \delta WAam \delta Wahet \delta WAhet \Omega MM^2 + 2 \delta WAAm \delta Wahet \delta WAhet \Omega MM^2 -$
$\delta Wahet^2 \delta WAhet \Omega MM^2 + 2 \delta WAAf \delta WAhet^2 \Omega MM^2 - 2 \delta Waam \delta WAhet^2 \Omega MM^2 +$
$2 \delta WAam \delta WAhet^2 \Omega MM^2 - \delta Wahet \delta WAhet^2 \Omega MM^2 + \delta WAhet^3 \Omega MM^2 -$
$2 \delta WAAf^2 \delta Wahom \Omega MM^2 + 4 \delta WAAf \delta Waam \delta Wahom \Omega MM^2 - 2 \delta WAAf \delta WAam \delta Wahom \Omega MM^2 -$
$2 \delta WAAf \delta WAAm \delta Wahom \Omega MM^2 + 2 \delta WAAf \delta Wahet \delta Wahom \Omega MM^2 -$
$2 \delta Waam \delta Wahet \delta Wahom \Omega MM^2 + 2 \delta WAam \delta Wahet \delta Wahom \Omega MM^2 -$
$4 \delta WAAf \delta WAhet \delta Wahom \Omega MM^2 + 2 \delta Waam \delta WAhet \delta Wahom \Omega MM^2 -$
$2 \delta WAam \delta WAhet \delta Wahom \Omega MM^2 + 2 \delta Wahet \delta WAhet \delta Wahom \Omega MM^2 -$
$2 \delta WAhet^2 \delta Wahom \Omega MM^2 + 2 \delta WAAf \delta Wahom^2 \Omega MM^2 - \delta Wahet \delta Wahom^2 \Omega MM^2 +$
$\delta WAhet \delta Wahom^2 \Omega MM^2 - 2 \delta WAAf \delta Waam \delta WAhom \Omega MM^2 + 2 \delta WAAf \delta WAam \delta WAhom \Omega MM^2 +$
$2 \delta WAAf \delta Wahet \delta WAhom \Omega MM^2 - 2 \delta WAam \delta Wahet \delta WAhom \Omega MM^2 +$
$2 \delta WAAm \delta Wahet \delta WAhom \Omega MM^2 - 2 \delta Wahet^2 \delta WAhom \Omega MM^2 +$
$2 \delta WAam \delta WAhet \delta WAhom \Omega MM^2 - 2 \delta WAAm \delta WAhet \delta WAhom \Omega MM^2 +$
$2 \delta Wahet \delta WAhet \delta WAhom \Omega MM^2 - 2 \delta WAAf \delta Wahom \delta WAhom \Omega MM^2 + \delta Wahet \delta WAhom^2 \Omega MM^2 -$
$\delta WAhet \delta WAhom^2 \Omega MM^2 + 2 \delta WAAf^2 \delta Waam \Omega MM^3 - 2 \delta WAAf \delta Waam^2 \Omega MM^3 -$
$2 \delta WAAf^2 \delta WAam \Omega MM^3 + 6 \delta WAAf \delta Waam \delta WAam \Omega MM^3 - 4 \delta WAAf \delta WAam^2 \Omega MM^3 -$
$2 \delta WAAf \delta Waam \delta WAAm \Omega MM^3 + 2 \delta WAAf \delta WAam \delta WAAm \Omega MM^3 + \delta WAAf^2 \delta Wahet \Omega MM^3 +$
$2 \delta WAAf \delta Waam \delta Wahet \Omega MM^3 - \delta Waam^2 \delta Wahet \Omega MM^3 + 2 \delta Waam \delta WAam \delta Wahet \Omega MM^3 -$
$2 \delta WAAf \delta WAAm \delta Wahet \Omega MM^3 - 2 \delta WAam \delta WAAm \delta Wahet \Omega MM^3 + \delta WAAm^2 \delta Wahet \Omega MM^3 +$
$2 \delta WAAf \delta Wahet^2 \Omega MM^3 + 2 \delta WAam \delta Wahet^2 \Omega MM^3 - 2 \delta WAAm \delta Wahet^2 \Omega MM^3 +$
$\delta Wahet^3 \Omega MM^3 - 3 \delta WAAf^2 \delta WAhet \Omega MM^3 + 4 \delta WAAf \delta Waam \delta WAhet \Omega MM^3 +$
$\delta Waam^2 \delta WAhet \Omega MM^3 - 8 \delta WAAf \delta WAam \delta WAhet \Omega MM^3 - 2 \delta Waam \delta WAam \delta WAhet \Omega MM^3 +$
$4 \delta WAAf \delta WAAm \delta WAhet \Omega MM^3 + 2 \delta WAam \delta WAAm \delta WAhet \Omega MM^3 - \delta WAAm^2 \delta WAhet \Omega MM^3 -$
$4 \delta WAAf \delta Wahet \delta WAhet \Omega MM^3 + 2 \delta Waam \delta Wahet \delta WAhet \Omega MM^3 -$
$4 \delta WAam \delta Wahet \delta WAhet \Omega MM^3 + 2 \delta WAAm \delta Wahet \delta WAhet \Omega MM^3 -$
$\delta Wahet^2 \delta WAhet \Omega MM^3 - 2 \delta WAAf \delta WAhet^2 \Omega MM^3 - 2 \delta Waam \delta WAhet^2 \Omega MM^3 +$
$2 \delta WAam \delta WAhet^2 \Omega MM^3 - \delta Wahet \delta WAhet^2 \Omega MM^3 + \delta WAhet^3 \Omega MM^3 +$
$2 \delta WAAf^2 \delta Wahom \Omega MM^3 - 4 \delta WAAf \delta Waam \delta Wahom \Omega MM^3 + 6 \delta WAAf \delta WAam \delta Wahom \Omega MM^3 -$
$2 \delta WAAf \delta WAAm \delta Wahom \Omega MM^3 + 2 \delta WAAf \delta Wahet \delta Wahom \Omega MM^3 -$
$2 \delta Waam \delta Wahet \delta Wahom \Omega MM^3 + 2 \delta WAam \delta Wahet \delta Wahom \Omega MM^3 +$
$4 \delta WAAf \delta WAhet \delta Wahom \Omega MM^3 + 2 \delta Waam \delta WAhet \delta Wahom \Omega MM^3 -$
$2 \delta WAam \delta WAhet \delta Wahom \Omega MM^3 + 2 \delta Wahet \delta WAhet \delta Wahom \Omega MM^3 -$
$2 \delta WAhet^2 \delta Wahom \Omega MM^3 - 2 \delta WAAf \delta Wahom^2 \Omega MM^3 - \delta Wahet \delta Wahom^2 \Omega MM^3 +$
$\delta WAhet \delta Wahom^2 \Omega MM^3 - 2 \delta WAAf \delta Waam \delta WAhom \Omega MM^3 + 2 \delta WAAf \delta WAam \delta WAhom \Omega MM^3 -$

$2\,\delta WAAf\,\delta Wahet\,\delta WAhom\,\Omega MM^3 - 2\,\delta WAam\,\delta Wahet\,\delta WAhom\,\Omega MM^3 +$
$2\,\delta WAAm\,\delta Wahet\,\delta WAhom\,\Omega MM^3 - 2\,\delta Wahet^2\,\delta WAhom\,\Omega MM^3 +$
$4\,\delta WAAf\,\delta WAhet\,\delta WAhom\,\Omega MM^3 + 2\,\delta WAam\,\delta WAhet\,\delta WAhom\,\Omega MM^3 -$
$2\,\delta WAAm\,\delta WAhet\,\delta WAhom\,\Omega MM^3 + 2\,\delta Wahet\,\delta WAhet\,\delta WAhom\,\Omega MM^3 -$
$2\,\delta WAAf\,\delta Wahom\,\delta WAhom\,\Omega MM^3 + \delta Wahet\,\delta WAhom^2\,\Omega MM^3 -$
$\delta WAhet\,\delta WAhom^2\,\Omega MM^3 - 4\,\delta WAaf^2\,(-2 + \Omega MM)\,\Omega MM\,(3\,\delta WAAf\,(-2 + \Omega MM) -$
$2\,(\delta Waam - 2\,\delta WAam + \delta WAAm - \delta Wahet - \delta WAhet + \delta Wahom + \delta WAhom)\,\Omega MM) -$
$\delta Waaf^2\,(-2 + \Omega MM)\,\big(2\,\delta Wahet + 2\,\delta WAhet - 4\,\delta WAhom + 8\,\delta WAaf\,\Omega MM -$
$8\,\delta WAAf\,\Omega MM + \delta Wahet\,\Omega MM + \delta WAhet\,\Omega MM - 2\,\delta WAhom\,\Omega MM - 4\,\delta WAaf\,\Omega MM^2 +$
$4\,\delta WAAf\,\Omega MM^2 + 3\,\delta Wahet\,\Omega MM^2 - \delta WAhet\,\Omega MM^2 - 2\,\delta WAhom\,\Omega MM^2 +$
$2\,\delta WAam\,\big(2 + \Omega MM + \Omega MM^2\big) - 2\,\delta WAAm\,\big(2 + \Omega MM + \Omega MM^2\big)\big) + 2\,\delta WAaf$
$\big(2\,\delta WAAf^2\,(-2 + \Omega MM)^2\,\Omega MM - \delta WAAf\,(-2 + \Omega MM)\,\big(2\,\delta Wahom - 2\,\delta WAhom + \delta Wahom\,\Omega MM -$
$\delta WAhom\,\Omega MM - 8\,\delta WAam\,\Omega MM^2 - 2\,\delta Wahet\,\Omega MM^2 - 6\,\delta WAhet\,\Omega MM^2 + 5\,\delta Wahom\,\Omega MM^2 +$
$3\,\delta WAhom\,\Omega MM^2 + \delta WAAm\,\big(-2 - \Omega MM + 3\,\Omega MM^2\big) + \delta Waam\,\big(2 + \Omega MM + 5\,\Omega MM^2\big)\big) +$
$\Omega MM\,\big(-2\,\delta Wahet\,\delta Wahom + 2\,\delta WAhet\,\delta Wahom - 2\,\delta Wahom^2 + 2\,\delta Wahet\,\delta WAhom -$
$2\,\delta WAhet\,\delta WAhom + 4\,\delta Wahom\,\delta WAhom - 2\,\delta WAhom^2 - \delta Wahet\,\delta Wahom\,\Omega MM +$
$\delta WAhet\,\delta Wahom\,\Omega MM - \delta Wahom^2\,\Omega MM + \delta Wahet\,\delta WAhom\,\Omega MM -$
$\delta WAhet\,\delta WAhom\,\Omega MM + 2\,\delta Wahom\,\delta WAhom\,\Omega MM - \delta WAhom^2\,\Omega MM + 4\,\delta WAam^2\,\Omega MM^2 +$
$4\,\delta WAam\,\delta Wahet\,\Omega MM^2 + 4\,\delta WAam\,\delta WAhet\,\Omega MM^2 + 4\,\delta Wahet\,\delta WAhet\,\Omega MM^2 -$
$4\,\delta WAam\,\delta Wahom\,\Omega MM^2 - 3\,\delta Wahet\,\delta Wahom\,\Omega MM^2 - \delta WAhet\,\delta Wahom\,\Omega MM^2 +$
$\delta Wahom^2\,\Omega MM^2 - 4\,\delta WAam\,\delta WAhom\,\Omega MM^2 - \delta Wahet\,\delta WAhom\,\Omega MM^2 -$
$3\,\delta WAhet\,\delta WAhom\,\Omega MM^2 + 2\,\delta Wahom\,\delta WAhom\,\Omega MM^2 + \delta WAhom^2\,\Omega MM^2 +$
$\delta Waam^2\,\big(-2 - \Omega MM + \Omega MM^2\big) + \delta WAAm^2\,\big(-2 - \Omega MM + \Omega MM^2\big) + \delta Waam\,\big(2\,\delta WAhet -$
$4\,\delta Wahom + 4\,\delta WAhom + \delta WAhet\,\Omega MM - 2\,\delta Wahom\,\Omega MM + 2\,\delta WAhom\,\Omega MM -$
$4\,\delta WAam\,\Omega MM^2 - \delta WAhet\,\Omega MM^2 + 2\,\delta Wahom\,\Omega MM^2 + 2\,\delta WAhom\,\Omega MM^2 +$
$2\,\delta WAAm\,\big(2 + \Omega MM + \Omega MM^2\big) - \delta Wahet\,\big(2 + \Omega MM + 3\,\Omega MM^2\big)\big) - \delta WAAm\,\big(4\,\delta WAhom +$
$2\,\delta WAhom\,\Omega MM + 4\,\delta WAam\,\Omega MM^2 - 2\,\delta WAhom\,\Omega MM^2 + \delta Wahet\,\big(-2 - \Omega MM + \Omega MM^2\big) -$
$2\,\delta Wahom\,\big(2 + \Omega MM + \Omega MM^2\big) + \delta WAhet\,\big(2 + \Omega MM + 3\,\Omega MM^2\big)\big)\big)\big) -$
$2\,\delta Waaf\,\big(6\,\delta WAaf^2\,(-2 + \Omega MM)^2\,\Omega MM + 2\,\delta WAAf^2\,(-2 + \Omega MM)^2\,\Omega MM -$
$\delta WAAf\,(\delta Waam - 2\,\delta WAam + \delta WAAm - \delta Wahet - \delta WAhet + \delta Wahom + \delta WAhom)$
$\big(4 - 7\,\Omega MM^2 + 3\,\Omega MM^3\big) + \delta WAaf\,(-2 + \Omega MM)$
$\big(-2\,\delta Wahom + 2\,\delta WAhom + 16\,\delta WAAf\,\Omega MM - \delta Wahom\,\Omega MM + \delta WAhom\,\Omega MM - 8\,\delta WAAf$
$\Omega MM^2 - 8\,\delta WAam\,\Omega MM^2 - 6\,\delta Wahet\,\Omega MM^2 - 2\,\delta WAhet\,\Omega MM^2 + 3\,\delta Wahom\,\Omega MM^2 +$
$5\,\delta WAhom\,\Omega MM^2 + \delta Waam\,\big(-2 - \Omega MM + 3\,\Omega MM^2\big) + \delta WAAm\,\big(2 + \Omega MM + 5\,\Omega MM^2\big)\big) +$
$\Omega MM\,\big(-2\,\delta WAAm^2 + 4\,\delta WAAm\,\delta Wahet - 2\,\delta Wahet^2 - 2\,\delta WAAm\,\delta WAhet +$
$2\,\delta WAhet^2 + 2\,\delta WAAm\,\delta Wahom - 2\,\delta WAhet\,\delta Wahom - 4\,\delta WAAm\,\delta WAhom +$
$4\,\delta Wahet\,\delta WAhom - 2\,\delta WAhet\,\delta WAhom + 2\,\delta Wahom\,\delta WAhom - 2\,\delta WAhom^2 -$
$\delta WAAm^2\,\Omega MM + 2\,\delta WAAm\,\delta Wahet\,\Omega MM - \delta Wahet^2\,\Omega MM - \delta WAAm\,\delta WAhet\,\Omega MM +$
$\delta WAhet^2\,\Omega MM + \delta WAAm\,\delta Wahom\,\Omega MM - \delta WAhet\,\delta Wahom\,\Omega MM - 2\,\delta WAAm\,\delta WAhom\,\Omega MM +$
$2\,\delta Wahet\,\delta WAhom\,\Omega MM - \delta WAhet\,\delta WAhom\,\Omega MM + \delta Wahom\,\delta WAhom\,\Omega MM - \delta WAhom^2$
$\Omega MM + 2\,\delta WAam^2\,\Omega MM^2 + \delta WAAm^2\,\Omega MM^2 - 2\,\delta WAAm\,\delta Wahet\,\Omega MM^2 + \delta Wahet^2\,\Omega MM^2 -$
$\delta WAAm\,\delta WAhet\,\Omega MM^2 + 2\,\delta Wahet\,\delta WAhet\,\Omega MM^2 - \delta WAhet^2\,\Omega MM^2 + \delta WAAm\,\delta Wahom$
$\Omega MM^2 - 2\,\delta Wahet\,\delta Wahom\,\Omega MM^2 + \delta WAhet\,\delta Wahom\,\Omega MM^2 + 2\,\delta WAAm\,\delta WAhom\,\Omega MM^2 -$
$2\,\delta Wahet\,\delta WAhom\,\Omega MM^2 - \delta WAhet\,\delta WAhom\,\Omega MM^2 + \delta Wahom\,\delta WAhom\,\Omega MM^2 +$
$\delta WAhom^2\,\Omega MM^2 + \delta Waam\,\big(-2\,\delta WAhet + 2\,\delta WAhom - \delta WAhet\,\Omega MM + \delta WAhom\,\Omega MM -$

```
2 δWahet ΩMM^2 + δWAhet ΩMM^2 + δWAhom ΩMM^2 - δWAam (2 + ΩMM + ΩMM^2) +
δWAAm (2 + ΩMM + ΩMM^2)) + δWAam (4 δWAhet - 2 δWahom + 2 δWAhom +
2 δWAhet ΩMM - δWahom ΩMM + δWAhom ΩMM - δWahom ΩMM^2 - 3 δWAhom ΩMM^2 +
δWAAm (2 + ΩMM - 3 ΩMM^2) + δWahet (-4 - 2 ΩMM + 4 ΩMM^2)))))) /
((1 + ΩMM)^3 (2 δWAAf - δWaaf (-2 + ΩMM) + 2 δWAaf (-2 + ΩMM) - δWAAf ΩMM +
δWaam ΩMM - 2 δWAam ΩMM + δWAAm ΩMM - δWahet ΩMM -
δWAhet ΩMM + δWahom ΩMM + δWAhom ΩMM)^2)}}
```

Internal Stability Conditions

First, we get the jacobian matrix when M is fixed. The solution for the characteristic polynomial at equilibria q=0 and q=1 give the eigenvalues that determine instability of these equilibria.

```
In[ ]:= jacobMfixed =
  Transpose[Map[D[recursionsMfixed, #] &, genotypesMfixed]] /. qFreplace /.
   x[{i_, j_}] → 0;
```

```
In[ ]:= charpoly0 = Det[(jacobMfixed /. q → 0 /. F → 0 /. μ → 0 // Simplify) -
      IdentityMatrix[Length[genotypesMfixed]] * λ] // Simplify;
charpoly1 = Det[(jacobMfixed /. q → 1 /. F → 0 /. μ → 0 // Simplify) -
      IdentityMatrix[Length[genotypesMfixed]] * λ] // Simplify;
```

We can get the stability conditions assuming weak selection using the weaksel substitution.

```
In[ ]:= δλ1sol0 =
  δλ1 /. Solve[Normal[Series[charpoly0 /. fitnessDiffs /. weaksel /. dg[1] → 1 /.
         dg[0] → 0 /. λ → 1 + δλ1 * ϵ, {ϵ, 0, 1}]] == 0, δλ1][[1]]
δλ1sol1 =
  δλ1 /. Solve[Normal[Series[charpoly1 /. fitnessDiffs /. weaksel /. dg[1] → 1 /.
         dg[0] → 0 /. λ → 1 + δλ1 * ϵ, {ϵ, 0, 1}]] == 0, δλ1][[1]]
```

```
Out[ ]= 1/(2 (1 + ΩMM))
  (2 δWaaf - 2 δWAAf - 3 δWaaf ΩMM + 4 δWAaf ΩMM - δWAAf ΩMM + δWaam ΩMM - δWAAm ΩMM +
    δWahet ΩMM - δWAhet ΩMM + δWahom ΩMM - δWAhom ΩMM + δWaaf ΩMM^2 - 2 δWAaf ΩMM^2 +
    δWAAf ΩMM^2 - δWaam ΩMM^2 + 2 δWAam ΩMM^2 - δWAAm ΩMM^2 +
    δWahet ΩMM^2 + δWAhet ΩMM^2 - δWahom ΩMM^2 - δWAhom ΩMM^2)
```

```
Out[ ]= 1/(2 (1 + ΩMM))
  (-2 δWaaf + 2 δWAAf - δWaaf ΩMM + 4 δWAaf ΩMM - 3 δWAAf ΩMM - δWaam ΩMM + δWAAm ΩMM -
    δWahet ΩMM + δWAhet ΩMM - δWahom ΩMM + δWAhom ΩMM + δWaaf ΩMM^2 - 2 δWAaf ΩMM^2 +
    δWAAf ΩMM^2 - δWaam ΩMM^2 + 2 δWAam ΩMM^2 - δWAAm ΩMM^2 +
    δWahet ΩMM^2 + δWAhet ΩMM^2 - δWahom ΩMM^2 - δWAhom ΩMM^2)
```

These can be re-written as follows

```
In[ ]:= δλ1sol0;
        (((1 - ΩMM/2) ((1 - F) δWAaf + F δWaaf - δWAAf ) +
              ΩMM/2 ((1 - F) δWAam + F δWaam - δWAAm + δWahet - δWAhom + F (δWahom - δWAhet))) /.
            F → (1 - ΩMM)/(1 + ΩMM)) - % // Simplify
        δλ1sol1;
        (((1 - ΩMM/2) ((1 - F) δWAaf + F δWAAf - δWaaf ) +
              ΩMM/2 ((1 - F) δWAam + F δWAAm - δWaam + δWAhet - δWahom + F (δWAhom - δWahet))) /.
            F → (1 - ΩMM)/(1 + ΩMM)) - % // Simplify

Out[ ]= 0

Out[ ]= 0
```

We can also see that the equilibria can be written in terms of these instability conditions.

```
In[ ]:= q /. qsol[[3]] // Factor;
        δλ1sol0/(δλ1sol0 + δλ1sol1) // Simplify;
        %% / % // Factor

Out[ ]= 1
```

Deleterious Mutations (equilibrium maintained at mutation-selection balance)

Equilibrium (mut-sel) - weak selection

Here, we will assume weak selection and very weak mutation. Giving a leading order solution for the inbreeding coefficient F. We are interested in the solution where q is around 0.

```
In[ ]:= Normal[Series[
            (solveme /. fitnessDiffs /. weaksel /. μ → μ * ϵ^3 //. q → q1 * ϵ), {ϵ, 0, 1}]];
        F0solMS = Flatten[Solve[% == 0, F]]

Out[ ]= {F → -(-1 + ΩMM)/(1 + ΩMM)}

In[ ]:= Normal[Series[(solveme /. fitnessDiffs /. weaksel /. μ → μ * ϵ^3 /.
                F → (F /. F0solMS) + δF * ϵ /. q → q1 * ϵ), {ϵ, 0, 2}]];
        Simplify[Flatten[Solve[% == 0, {q1, δF}], 1]]

        ••• Solve : Equations may not give solutions for all "solve" variables.

Out[ ]= {q1 → 0}
```

In[•]:=
```
Normal[Series[(solveme /. fitnessDiffs /. weaksel /. μ → μ * ϵ^3 /.
      F → (F /. F0solMS) + δF * ϵ /. q → q2 * ϵ^2), {ϵ, 0, 3}]];
qsolMS = Simplify[Flatten[Solve[% == 0, {q2, δF}], 1]]
```

Out[•]=

$$\Big\{q2 \to (2\,\mu\,(1+\Omega MM)) \Big/ \Big(\Omega MM\,(\delta WAAm - \delta Wahet + \delta WAhet - \delta Wahom + \delta WAhom + 2\,\delta WAaf\,(-2+\Omega MM) + \delta Waam\,(-1+\Omega MM) - 2\,\delta WAam\,\Omega MM + \delta WAAm\,\Omega MM - \delta Wahet\,\Omega MM - \delta WAhet\,\Omega MM + \delta Wahom\,\Omega MM + \delta WAhom\,\Omega MM) + \delta WAAf\,(2+\Omega MM-\Omega MM^2) - \delta Waaf\,(2-3\,\Omega MM+\Omega MM^2)\Big),$$
$$\delta F \to \frac{(-1+\Omega MM)\,(2\,\delta WAAf + 2\,\delta Waaf\,(-1+\Omega MM) + (-2\,\delta WAaf - \delta Wahet + \delta WAhet)\,\Omega MM)}{(1+\Omega MM)^2}\Big\}$$

As above, this can be written in terms of the advantage (or in this case disadvantage) of the a allele when it is rare

In[•]:=

$$Ia = \left(\left(\left(1-\frac{\Omega MM}{2}\right)((1-F)\,\delta WAaf + F\,\delta Waaf - \delta WAAf) + \frac{\Omega MM}{2}((1-F)\,\delta WAam + F\,\delta Waam - \delta WAAm + \delta Wahet - \delta WAhom + F\,(\delta Wahom - \delta WAhet))\right) /.\ F \to \frac{1-\Omega MM}{1+\Omega MM}\right);$$

```
q2 /. qsolMS;
```

$$\frac{\mu}{-Ia};$$

```
%% - % // Simplify
```

Out[•]= 0

Mutation Load

Here, we are going to assume that fitness effects of multiple loci are multiplicative.
Following Charlesworth & Charlesworth (1992), fitness with j homozygotes and i heterozygotes is
$w_{ij} = (1-s)^j\,(1-h\,s)^i$.
Or
$w_{ij} = (Waa)^j\,(WAa)^i\,(WAA)^{1-i-j}$ in our notation.
Assuming we have l loci that are unlinked, the expected values for j and i are just the expected genotype frequencies based on the allele frequency. We assume a fraction k of loci experience selection in male gametes and the other loci experience only diploid selection.

```
In[•]:= freqaa1 = (q1^2 (1 - F) + q1 F);
freqAa1 = (2 q1 (1 - q1) (1 - F));
freqAA1 = ((1 - q1)^2 (1 - F) + F (1 - q1));
freqaa2 = (q2^2 (1 - F) + q2 F);
freqAa2 = (2 q2 (1 - q2) (1 - F));
freqAA2 = ((1 - q2)^2 (1 - F) + F (1 - q2));
meanFitMS = Normal[Series[(Waa)^(l (k freqaa1+(1-k) freqaa2))
      (WAa)^(l (k freqAa1 + (1-k) freqAa2)) (WAA)^(l (k freqAA1 + (1-k) freqAA2)) /. q1 → (μ * ϵμ)/(-δλ1 * ϵ) /.
     q2 → ((μ * ϵμ)/(-δλ1nohapsel * ϵ)) /. noSexDiff /. fitnessDiffs /. weaksel,
   {ϵ, 0, 2}, {ϵμ, 0, 1}]] /. ϵ → 1 /. ϵμ → 1 // Simplify
```

$$\text{Out[•]= } e^{\frac{l\,(2\,(\delta WAa-\delta WAA)+F\,(\delta Waa-2\,\delta WAa+\delta WAA))\,((-1+k)\,\delta\lambda 1-k\,\delta\lambda 1nohapsel)\,\mu}{\delta\lambda 1\,\delta\lambda 1nohapsel}}$$

This is written in a simplified form in the appendix

```
In[•]:= fitTerm = ((2 - F) ( δWAA) - 2 (1 - F) (δWAa ) - F (δWaa ));
Exp[-l fitTerm ( k q + (1 - k) q2 ) ] - meanFitMS /. δλ1 → -μ / q /.
  δλ1nohapsel → -μ / q2 // Simplify

Out[•]= 0
```

Inbreeding Depression

Inbreeding depression is given by δ=1-wbars/wbaro, where wbars and wbaro are the average fitnesses of offspring produced by selfing and outcrossing, respectively.

To calculate inbreeding depression, we must get the frequency of the different genotypes in offspring produced by outcrossing or selfing.

```
In[•]:= selffreqaa1 = ((q1^2 (1 - F) + q1 F) + ((2 q1 (1 - q1) (1 - F)) / 4));
selffreqAa1 = (q1 (1 - q1) (1 - F));
selffreqAA1 = ((1 - q1)^2 (1 - F) + F (1 - q1) + (((2 q1 (1 - q1) (1 - F)) / 4)));
selffreqaa2 = ((q2^2 (1 - F) + q2 F) + ((2 q2 (1 - q2) (1 - F)) / 4));
selffreqAa2 = (q2 (1 - q2) (1 - F));
selffreqAA2 = ((1 - q2)^2 (1 - F) + F (1 - q2) + (((2 q2 (1 - q2) (1 - F)) / 4)));
```

In[∘]:=
$$\text{outfreqaa1} = \text{freqaa1}\,(\text{freqaa1} + \text{freqAa1}) + \frac{\text{freqAa1}^2}{4};$$
$$\text{outfreqAa1} = \left(\text{freqaa1 freqAa1} + \text{freqAA1 freqAa1} + \frac{\text{freqAa1}^2}{2} + 2\,\text{freqAA1 freqaa1}\right);$$
$$\text{outfreqAA1} = \left(\text{freqAA1}\,(\text{freqAA1} + \text{freqAa1}) + \frac{\text{freqAa1}^2}{4}\right);$$
$$\text{outfreqaa2} = \text{freqaa2}\,(\text{freqaa2} + \text{freqAa2}) + \frac{\text{freqAa2}^2}{4};$$
$$\text{outfreqAa2} = \left(\text{freqaa2 freqAa2} + \text{freqAA2 freqAa2} + \frac{\text{freqAa2}^2}{2} + 2\,\text{freqAA2 freqaa2}\right);$$
$$\text{outfreqAA2} = \left(\text{freqAA2}\,(\text{freqAA2} + \text{freqAa2}) + \frac{\text{freqAa2}^2}{4}\right);$$

We then get wbars and wbaro due to deleterious mutations at mutation selection balance.

In[∘]:=
$$\text{wbars} = (\text{Waa})^{l\,(k\,\text{selffreqaa1}+(1-k)\,\text{selffreqaa2})}\,(\text{WAa})^{l\,(k\,\text{selffreqAa1}+(1-k)\,\text{selffreqAa2})}\,(\text{WAA})^{l\,(k\,\text{selffreqAA1}+(1-k)\,\text{selffreqAA2})};$$
$$\text{wbaro} = (\text{Waa})^{l\,(k\,\text{outfreqaa1}+(1-k)\,\text{outfreqaa2})}\,(\text{WAa})^{l\,(k\,\text{outfreqAa1}+(1-k)\,\text{outfreqAa2})}\,(\text{WAA})^{l\,(k\,\text{outfreqAA1}+(1-k)\,\text{outfreqAA2})};$$

We approximate the ratio of selfing mean fitness to outcrossing mean fitness using the same approximations used to calculate mean fitness above (weak selection and migration, calculated in sequential Taylor Series).
We re-write this result in the form presented in the appendix.

In[∘]:=
$$\text{Normal}\Big[\text{Series}\Big[\frac{\text{wbars}}{\text{wbaro}} \;/.\; \text{q1} \to \frac{\mu\ *\epsilon\mu}{-\delta\lambda 1 * \epsilon} \;/.\; \text{q2} \to \left(\frac{\mu\ *\epsilon\mu}{-\delta\lambda\text{1nohapsel} * \epsilon}\right) \;/.\; \text{noSexDiff} \;/.\; \text{fitnessDiffs} \;/.\; \text{weaksel}, \{\epsilon, 0, 2\}, \{\epsilon\mu, 0, 1\}\Big]\Big] \;/.\; \epsilon \to 1 \;/.\; \epsilon\mu \to 1 \;//\; \text{Simplify};$$
$$\text{Exp}\left[-l\left(-\text{sbar}\delta\mu\left(k\,\frac{\mu}{-\delta\lambda 1} + (1-k)\,\frac{\mu}{-\delta\lambda\text{1nohapsel}}\right)\right)\right] \;/.\; \text{sbar}\delta\mu \to \frac{(1+F)}{2}\,(\delta\text{WAA} + \delta\text{Waa} - 2\,\delta\text{WAa});$$
```
% - %% // Simplify
```

Out[∘]= 0

We also consider inbreeding depression when some loci experience balancing selection.

In[∘]:=
$$\text{outBS} = (\text{Waa})^{l2\,(\text{outfreqaa2})}\,(\text{WAa})^{l2\,(\text{outfreqAa2})}\,(\text{WAA})^{l2\,(\text{outfreqAA2})};$$
$$\text{selfBS} = (\text{Waa})^{l2\,\text{selffreqaa2}}\,(\text{WAa})^{l2\,\text{selffreqAa2}}\,(\text{WAA})^{l2\,\text{selffreqAA2}};$$

Calculating the ratio between self and outcross fitness to leading order with weak selection and rewriting gives:

```
In[]:= Normal[Series[selfBS/outBS /. noSexDiff /. fitnessDiffs /. weaksel, {ϵ, 0, 1}]] /. ϵ → 1 //
         Simplify;
       1 + l2 q2 (1 - q2) (sbarδμ) /. sbarδμ → (1 + F)/2 (δWAA + δWaa - 2 δWAa) // Simplify;
       % - %% // Simplify
Out[]= 0
```

Evolution of selfing rate

General

We now calculate the external stability of the M-fixed equilibria by looking at the recursion equations involving the m allele and assuming that it is rare.

```
In[]:= malleles = Map[(Mgeno[#[[1]]] == 2 || Mgeno[#[[2]]] == 2) &, genotypes];
```

```
In[]:= recursionsExt = Pick[newkid, malleles];
       genotypesExt = Map[x, Pick[genotypes, malleles]];
       differenceExt = recursionsExt - genotypesExt;
       jacobExt =
         Factor[Transpose[Map[D[recursionsExt, #] &, genotypesExt]] /. qFreplace /.
           x[{i_, j_}] → 0];
```

The leading eigenvalue can be found by solving the following characteristic polynomial for λ:

```
In[]:= charpoly = Det[IdentityMatrix[7] * λ - jacobExt];
```

Unlike the polyandry case, we have to consider the full jacobian because m homozygotes can occur through selfing even when m is rare.

If the m allele increases in frequency when rare (λ>1), then it invades and the selfing rate evolves.

Modifier evolution with balancing selection - weak dominant modifier

```
In[]:= Normal[Series[charpoly /. fitnessDiffs /. weaksel /. μ → 0 /. weakΩmod /.
           F → (F /. Fsol0), {ϵ, 0, 0}]];
       Solve[% == 0, λ] // Flatten
```

Out[]=
$$\left\{\lambda \to 1,\ \lambda \to \frac{1-\Omega MM}{2},\ \lambda \to \frac{1-\Omega MM}{2},\ \lambda \to \frac{1-\Omega MM}{2},\ \lambda \to -\frac{1}{2}\left(1-2\,r+2\,r^2\right)(-1+\Omega MM),\right.$$
$$\lambda \to \frac{1}{4}\left(3-2\,r-\Omega MM-\sqrt{1+4\,r+4\,r^2+2\,\Omega MM-12\,r\,\Omega MM+\Omega MM^2}\right),$$
$$\left.\lambda \to \frac{1}{4}\left(3-2\,r-\Omega MM+\sqrt{1+4\,r+4\,r^2+2\,\Omega MM-12\,r\,\Omega MM+\Omega MM^2}\right)\right\}$$

In[ ]:=
```
Normal[
  Series[charpoly /. λ → 1 + δλ1 * ϵ /. fitnessDiffs /. weaksel /. μ → 0 /. weakΩmod /.
    F → (F /. Fsol0), {ϵ, 0, 1}]];
δλ1solExt = Flatten[Factor[Solve[% == 0, δλ1]]]
```

Out[ ]=
$$\left\{\delta\lambda 1 \to -\frac{(-1+c)\ (-\delta\Omega mm + \delta\Omega mm\ \Omega MM - 2\ \delta\Omega Mm\ \Omega MM)}{2\ (1+\Omega MM)\ (1-c+c\ \Omega MM)}\right\}$$

If the modifier increases the outcrossing rate (0<δΩMm<δΩmm), then this is always negative because selfing has a transmission advantage when c=/=1. The fact that δλ1 is negative can be seen clearly in the following form:

In[ ]:=
```
(δλ1 /. δλ1solExt);
-(1 - c) / (2 (1 - c (1 - ΩMM))) (δΩMm + (δΩmm - δΩMm) F) - % /. Fsol0 // Simplify
```

Out[ ]= 0

In[ ]:=
```
(δΩMm + (δΩmm - δΩMm) F)
% /. δΩMm → ΩMm - ΩMM /. δΩmm → Ωmm - ΩMM // Simplify
(1 - F) (ΩMm - ΩMM) + F (Ωmm - ΩMM) - % // Simplify
```

Out[ ]= $F\ (\delta\Omega mm - \delta\Omega Mm) + \delta\Omega Mm$

Out[ ]= $F\ (\Omega mm - \Omega Mm) + \Omega Mm - \Omega MM$

Out[ ]= 0

We can next assume that c is near 1 and that the modifier is dominant.

In[ ]:=
```
Normal[Series[
   (charpoly /. λ → 1 + δλ2 * ϵ^2
          /. c → 1
         /. fitnessDiffs
        /. weaksel
       /. μ → 0
      /. F → (F /. Fsol0) + (δF) * ϵ
     /. weakΩmod /. δΩmm → δΩMm),
   {ϵ, 0, 2}]];
δλ2solExt = Flatten[Solve[% == 0, δλ2]];
```

This is rearranged by first dividing by q(1-q), and then inputting the equilibrium solution.

In[ ]:=
```
δλ2solExtRearranged =
  (Factor[(δλ2 /. δλ2solExt) / (q (1 - q))] /. qsol[[3]]) // Simplify
```

Out[ ]=
$$\frac{1}{(1+\Omega MM)^2}\delta\Omega Mm\ (-2\ \delta WAAf + \delta Waaf\ (-2+\Omega MM) - 2\ \delta WAaf\ (-2+\Omega MM) + \delta WAAf\ \Omega MM - \delta Waam\ \Omega MM + 2\ \delta WAam\ \Omega MM - \delta WAAm\ \Omega MM + \delta Wahet\ \Omega MM + \delta WAhet\ \Omega MM - \delta Wahom\ \Omega MM - \delta WAhom\ \Omega MM)$$

This can be re-written in terms of the invasion conditions.

In[◦]:=
```
q (1 - q) δλ2solExtRearranged;
q (1 - q) δΩMm / ((1 + ΩMM) ΩMM) ((δλ0 + δλ1)) /. δλ0 → (δλ1sol0) /. δλ1 → (δλ1sol1) //
   Simplify;
% - %% // Simplify
```
Out[◦]= 0

Modifier evolution with mutation selection balance - weak dominant modifier

In[◦]:=
```
Normal[Series[charpoly /. μ → ϵ^3 μ /. fitnessDiffs /. weaksel /. weakΩmod /.
      F → (F /. F0solMS) /. q → q2 * ϵ^2, {ϵ, 0, 0}]];
Solve[% == 0, λ] // Flatten
```
Out[◦]=
$$\left\{\lambda \to 1,\ \lambda \to \frac{1-\Omega MM}{2},\ \lambda \to \frac{1-\Omega MM}{2},\ \lambda \to \frac{1-\Omega MM}{2},\ \lambda \to -\frac{1}{2}\left(1-2\,r+2\,r^2\right)(-1+\Omega MM),\right.$$
$$\lambda \to \frac{1}{4}\left(3-2\,r-\Omega MM-\sqrt{1+4\,r+4\,r^2+2\,\Omega MM-12\,r\,\Omega MM+\Omega MM^2}\right),$$
$$\left.\lambda \to \frac{1}{4}\left(3-2\,r-\Omega MM+\sqrt{1+4\,r+4\,r^2+2\,\Omega MM-12\,r\,\Omega MM+\Omega MM^2}\right)\right\}$$

In[◦]:=
```
Normal[Series[
    (charpoly /. λ → 1 + δλ1 * ϵ /. μ → ϵ^3 μ /. fitnessDiffs /. weaksel /. weakΩmod /.
       F → (F /. F0solMS) + δF * ϵ /. q → q2 * ϵ^2), {ϵ, 0, 1}]];
δλ1solExtMS = Flatten[Factor[Solve[% == 0, δλ1]]]
```
Out[◦]=
$$\left\{\delta\lambda 1 \to -\frac{(-1+c)\,(-\delta\Omega mm+\delta\Omega mm\,\Omega MM-2\,\delta\Omega Mm\,\Omega MM)}{2\,(1+\Omega MM)\,(1-c+c\,\Omega MM)}\right\}$$

We will now also assume that c is 1 and that the modifier is dominant

In[◦]:=
```
Normal[Series[
    (charpoly /. λ → 1 + δλ2 * ϵ^2 /. μ → ϵ^3 μ /. fitnessDiffs /. weaksel /. weakΩmod /.
         δΩmm → δΩMm /. F → (F /. F0solMS) + δF * ϵ /.
       q → q2 * ϵ^2 /. c → 1), {ϵ, 0, 2}]];
δλ2solExtMS = Flatten[Factor[Solve[% == 0, δλ2]]]
```
Out[◦]= {δλ2 → 0}

In[◦]:=
```
Normal[Series[
    (charpoly /. λ → 1 + δλ3 * ϵ^3 /. μ → ϵ^3 μ /. fitnessDiffs /. weaksel /. weakΩmod /.
         δΩmm → δΩMm /. F → (F /. F0solMS) + δF * ϵ /.
       q → q2 * ϵ^2 /. c → 1), {ϵ, 0, 3}]];
δλ3solExtMS = Flatten[Factor[Solve[% == 0, δλ3]]]
```
Out[◦]= {δλ3 → 0}

In[•]:=
```
Normal[Series[
    (charpoly /. λ → 1 + δλ4 * ϵ^4 /. μ → ϵ^3 μ /. fitnessDiffs /. weaksel /. weakΩmod /.
          δΩmm → δΩMm /. F → (F /. F0solMS) + δF * ϵ /.
        q → q2 * ϵ^2 /. c → 1), {ϵ, 0, 4}]];
δλ4solExtMS = Simplify[Flatten[Factor[Solve[% == 0, δλ4]]] /. qsolMS]
```

Out[•]=
```
{δλ4 →
  - ((2 δΩMm μ (-2 δWAAf + δWaaf (-2 + ΩMM) - 2 δWAaf (-2 + ΩMM) + δWAAf ΩMM - δWaam ΩMM +
          2 δWAam ΩMM - δWAAm ΩMM + δWahet ΩMM + δWAhet ΩMM - δWahom ΩMM - δWAhom ΩMM)) /
     ((1 + ΩMM) (-ΩMM (δWAAm - δWahet + δWAhet - δWahom + δWAhom +
              2 δWAaf (-2 + ΩMM) + δWaam (-1 + ΩMM) - 2 δWAam ΩMM + δWAAm ΩMM -
              δWahet ΩMM - δWAhet ΩMM + δWahom ΩMM + δWAhom ΩMM) +
          δWaaf (2 - 3 ΩMM + ΩMM^2) + δWAAf (-2 - ΩMM + ΩMM^2))))}
```

This is re-written in terms of the invasion conditions

In[•]:=
```
Factor[δλ4 /. δλ4solExtMS];
% * q / (q2 /. qsolMS) // Factor;
(δΩMm q)/((1 + ΩMM) ΩMM) ((δλ0 + δλ1)) /. δλ0 → (δλ1sol0) /. δλ1 → (δλ1sol1) // Simplify;
% - %% // Simplify
```

Out[•]= 0

Modifier growth rates in terms of inbreeding depression

The modifier growth rate with c=1 and weak dominant modifiers with balancing selection and mutation-selection balance, respectively, are

In[•]:=
```
eigenBS = q (1 - q) (δΩMm)/((1 + ΩMM) ΩMM) ((δλ0 + δλ1)) /. δλ0 → (δλ1sol0) /. δλ1 → (δλ1sol1);
eigenMS = q (δΩMm)/((1 + ΩMM) ΩMM) ((δλ0 + δλ1)) /. δλ0 → (δλ1sol0) /. δλ1 → (δλ1sol1);
```

When there are no sex differences in selection and haploid expression, these can be re-written according to the standard inbreeding depression calculated in Charlesworth and Charlesworth (1992).

In[•]:=
```
haploidExpression = {
    δWAhom → δWA,
    δWAhet → δWA,
    δWahet → δWa,
    δWahom → δWa};
IBD = 1 - wbars/wbaro;
```

Here, we take the leading order approximation of inbreeding depression according to our weak selection and mutation assumptions, then show that eigenMS and eigenBS are equivalent to δΩMm(1+F)IBD.

```
In[•]:= Normal[Series[IBD /. fitnessDiffs /. weaksel /. q1 → (μ * ϵ^3)/(-δλ1sol0 * ϵ) /. noSexDiff /.
          k → 1, {ϵ, 0, 3}]] /. ϵ → 1 // Simplify;
        δΩMm (1 + F) δ /. δ → % /. q → (q2 /. qsolMS) /. noSexDiff /. haploidExpression /.
           Fsol0 /. l → 1 // Simplify;
        eigenMS - % /. q → (q2 /. qsolMS) /. noSexDiff /. haploidExpression // Simplify
Out[•]= 0

In[•]:= Normal[Series[IBD /. fitnessDiffs /. noSexDiff /. weaksel, {ϵ, 0, 1}]] /. k → 1 /.
           ϵ → 1 /. q1 → q // Simplify;
        δΩMm (1 + F) δ /. δ → % /. noSexDiff /. haploidExpression /. Fsol0 /. l → 1 // Simplify;
        eigenBS - % /. noSexDiff /. haploidExpression // Simplify
Out[•]= 0
```

This is also re-written in the following terms.

```
In[•]:= Normal[Series[IBD /. fitnessDiffs /. weaksel /. q1 → (μ * ϵ^3)/(-δλ1sol0 * ϵ) /. noSexDiff,
            {ϵ, 0, 3}]] /. k → 1 /. l → 1 /. ϵ → 1 /. haploidExpression // Simplify;
        q2 * (-sbarδμ) /. qsolMS /. noSexDiff /. haploidExpression /.
           sbarδμ → (1 + F)/2 (δWAA + δWaa - 2 δWAa) // Simplify;
        % - %% // Simplify

        Normal[Series[IBD /. fitnessDiffs /. noSexDiff /. weaksel, {ϵ, 0, 1}]] /. l → 1 /.
            k → 1 /. ϵ → 1 /. haploidExpression // Simplify;
        q1 (1 - q1) * (-sbarδμ) /. qsolMS /. noSexDiff /. haploidExpression /.
           sbarδμ → (1 + F)/2 (δWAA + δWaa - 2 δWAa) // Simplify;
        % - %% // Simplify
Out[•]= 0

Out[•]= 0
```

In[ ]:=
```
Normal[Series[IBD /. fitnessDiffs /. weaksel /. q1 → μ * ϵ^3 / (-δλ1sol0 * ϵ) /. noSexDiff,
      {ϵ, 0, 3}]] /. k → 1 /. l → 1 /. ϵ → 1 /. haploidExpression // Simplify;
q2 * ((δλ0 + δλ1)) / (2 ΩMM) /. δλ0 → (δλ1sol0) /. δλ1 → (δλ1sol1) /. qsolMS /. noSexDiff /.
    haploidExpression /. sbarδμ → (1 + F)/2 (δWAA + δWaa - 2 δWAa) // Simplify;
% - %% /. Fsol0 // Simplify

Normal[Series[IBD /. fitnessDiffs /. noSexDiff /. weaksel, {ϵ, 0, 1}]] /. k → 1 /.
    l → 1 /. ϵ → 1 /. haploidExpression // Simplify;
q1 (1 - q1) * ((δλ0 + δλ1)) / (2 ΩMM) /. δλ0 → (δλ1sol0) /. δλ1 → (δλ1sol1) /. qsolMS /.
    noSexDiff /. haploidExpression /.
  sbarδμ → (1 + F)/2 (δWAA + δWaa - 2 δWAa) // Simplify;
% - %% /. Fsol0 // Simplify
```

Out[ ]= 0

Out[ ]= 0

If we use a modified calculation for mean fitness (called wstarbar) that includes male gametic fitness and weights male and female fitnesses according to the outcrossing rate, then the corresponding inbreeding depression (IBDstar) also gives eigenMS and eigenBS as δΩMm(1+F)IBD when allowing sex differences in selection or non-haploid gametic expression.

In[ ]:=
$$\text{wstarbars} = \left(\frac{\text{Waam Wahom }\Omega\text{MM} + \text{Waaf }(2-\Omega\text{MM})}{2}\right)^{l\,(k\,\text{selffreqaa1}+(1-k)\,\text{selffreqaa2})}\left(\frac{\text{WAam }\frac{(\text{WAhet}+\text{Wahet})}{2}\,\Omega\text{MM} + \text{WAaf }(2-\Omega\text{MM})}{2}\right)^{l\,(k\,\text{selffreqAa1}+(1-k)\,\text{selffreqAa2})}\left(\frac{\text{WAAm WAhom }\Omega\text{MM} + \text{WAAf }(2-\Omega\text{MM})}{2}\right)^{l\,(k\,\text{selffreqAA1}+(1-k)\,\text{selffreqAA2})};$$

$$\text{wstarbaro} = \left(\frac{\text{Waam Wahom }\Omega\text{MM} + \text{Waaf }(2-\Omega\text{MM})}{2}\right)^{l\,(k\,\text{outfreqaa1}+(1-k)\,\text{outfreqaa2})}\left(\frac{\text{WAam }\frac{(\text{WAhet}+\text{Wahet})}{2}\,\Omega\text{MM} + \text{WAaf }(2-\Omega\text{MM})}{2}\right)^{l\,(k\,\text{outfreqAa1}+(1-k)\,\text{outfreqAa2})}\left(\frac{\text{WAAm WAhom }\Omega\text{MM} + \text{WAAf }(2-\Omega\text{MM})}{2}\right)^{l\,(k\,\text{outfreqAA1}+(1-k)\,\text{outfreqAA2})};$$

In[ ]:=
$$\text{IBDstar} = 1 - \frac{\text{wstarbars}}{\text{wstarbaro}};$$

```
In[ ]:= Normal[Series[IBDstar /. fitnessDiffs /. weaksel /. q1 → (μ * ϵ^3)/(-δλ1sol0 * ϵ) /. k → 1,
        {ϵ, 0, 3}]] /. ϵ → 1 // Simplify;
    δΩMm (1 + F) δ /. δ → % /. q → (q2 /. qsolMS) /. Fsol0 /. l → 1 // Simplify;
    eigenMS - % /. q → (q2 /. qsolMS) // Simplify

Out[ ]= 0

In[ ]:= Normal[Series[IBDstar /. fitnessDiffs /. weaksel, {ϵ, 0, 1}]] /. k → 1 /. ϵ → 1 /.
      q1 → q // Simplify;
    δΩMm (1 + F) δ /. δ → % /. Fsol0 /. l → 1 // Simplify;
    eigenBS - % // Simplify

Out[ ]= 0
```
