# Supplementary material for: Gametic selection favours polyandry and selfing
Source: PLoS Genet. 2024 Feb 16;20(2):e1010660. doi: 10.1371/journal.pgen.1010660 (PMC10903963; doi:10.1371/journal.pgen.1010660)
Supplement: S1 Appendix — Detailed description of the recursion equations describing genotype frequency changes. (PDF) [file pgen.1010660.s004.pdf]

S1 Appendix: Two-Locus Model Details

In order to track mating events between diploids, we census the zygotic genotype frequencies (x_{ij}) at the **A** and **M** loci ($i, j \in \{MA, Ma, mA, ma\}$). We assume that maternal i and paternal j haplotypes are interchangeable resulting in a total of ten diploid genotypes, ij. To simplify notation, we use functions $f(ij) \in \{MM, Mm, mm\}$ to denote the **M** locus genotype and $g(ij) \in \{AA, Aa, aa\}$ to give the **A** locus genotype for ij diploids. These are simply indicator functions that give the one locus genotypes from both two-locus haplotypes. In species with separate sexes, we assume that the probability of developing as a male (♂) or female (♀) is independent of the genotype at the **A** and **M** loci. After selection, genotype frequencies for adults of sex $h \in \{♂, ♀\}$ are given by

$$\bar{x}_{ij}^{h\prime} = \left(1 + s_{g(ij)}^{h}\right) \bar{x}_{ij} / \sum_{i} \sum_{j} \left(1 + s_{g(ij)}^{h}\right) \bar{x}_{ij}. \tag{S1-1}$$

After selection among diploid adults, gametes are produced. Before mutation and selection, the frequency of gametes of genotype $k \in \{MA, Ma, mA, ma\}$ produced by adults of sex h with genotype ij is given by $y_{k,ij}^{h}$. Gametes inherit parental haplotypes ($y_{k,ij}^{h} = \bar{x}_{ij}^{h\prime}/2$ for $k = i$ and $k = j$), unless the parent is a double heterozygote (e.g., $i = MA$ and $j = ma$ or $i = Ma$ and $j = mA$). In double heterozygotes, we have to take account of the recombination rate between loci and $y_{k,ij}^{h} = \bar{x}_{ij}^{h\prime}(1-r)/2$ when $k = i$ or $k = j$ and $y_{k,ij}^{h} = \bar{x}_{ij}^{h\prime}(1-r)/2$ otherwise. No other gamete genotypes are possible, $y_{k,ij}^{h} = 0$ for all other combinations of i, j, k. We then assume that mutation from A to a occurs at rate μ. That is, after mutation, the gamete/gametophyte frequencies are $y_{k,ij}^{h\prime} = (1 - \mu)y_{k,ij}^{h}$ when $k = MA$ or $k = mA$ and $y_{Ma,ij}^{h\prime} = y_{Ma,ij}^{h} + \mu y_{MA,ij}^{h}$ and $y_{ma,ij}^{h\prime} = y_{ma,ij}^{h} + \mu y_{mA,ij}^{h}$. In short, we calculate the gamete/gametophyte genotype frequencies produced by adults of different genotypes after diploid selection and meiosis (with recombination and mutation).

The fitness of male gametes during competition depends on their **A**-locus genotype, indicated by $\alpha(k) \in \{A, a\}$), and the **A**-locus genotype of the father, given

by $g(ij)$ as above. That is, the fitness of a male gamete with genotype k produced by a male with genotype ij is $1 + s_{\alpha(k)}^{g(ij)}$. New mutations in AA males can produce a-bearing gametes with fitness $1 + s_{a}^{AA}$, but these are rare and do not feature in our results.

Male gametes compete under the mating system that is specified by the **M** locus. Under monandry or selfing, male gametes produced by a single individual compete with one another for fertilisation. In these matings that involve one male/hermaphrodite of genotype ij, the male gamete allele frequencies after haploid selection are

$$y_{k,ij}^{♂,o} = \left(1 + s_{\alpha(k)}^{g(ij)}\right) y_{k,ij}^{♂\prime} / \sum_{k} \left(1 + s_{\alpha(k)}^{g(ij)}\right) y_{k,ij}^{♂\prime}. \quad \text{(S1-2)}$$

Under polyandry or outcrossing, all male gametes compete in a common pool such that, after haploid selection, the frequency of male gametes with genotype k is

$$y_{k}^{♂,p} = \left(\sum_{i} \sum_{j} \left(1 + s_{\alpha(k)}^{g(ij)}\right) \pi_f y_{k,ij}^{♂\prime} \right) / \left(\sum_{k} \sum_{i} \sum_{j} \left(1 + s_{\alpha(k)}^{g(ij)}\right) \pi_f y_{k,ij}^{♂\prime} \right) \quad \text{(S1-3)}$$

where $\pi_f = 1 - c\left(1 - \Omega_f\right)$ accounts for 'pollen discounting' via parameter c. When $c = 1$, selfing results in a proportional decrease in the number of male gametes that are available for outcrossing. When $c = 0$, individuals donate the same number of male gametes to a common gamete pool for outcrossing, irrespective of their selfing rate. For polyandry, we assume that $c = 0$ because there is no selfing.

First, we assume that the **M** locus controls the degree of monandry/polyandry. Specifically, a fraction Π_f of the eggs/ovules produced by a mother with genotype $f(ij)$ at the **M** locus is mated polyandrously and the remaining fraction $(1 - \Pi_{f(ij)}$ is mated monandrously. Thus, we consider one hundred possible mating combinations between the ten female and male genotypes to get the zygotic genotype

frequencies in the next generation, given by

$$x'_{kl} = y^{♀\prime}_{l,ij}\Pi_{f(ij)}y^{♂,p}_{k} + y^{♀\prime}_{l,ij}\left(1 - \Pi_{f(ij)}\right)\left(\sum_{i}\sum_{j} y^{♂,o}_{k,ij}\right). \tag{S1-4}$$

Second, we assume that the **M** locus controls the degree of selfing versus outcrossing. Specifically, a fraction of eggs/ovules are specified to mate via outcrossing, $\Omega_{f(ij)}$, or selfing, $(1 - \Omega_{f(ij)})$. For example, a fraction $(1 - \Omega_{f(ij)})$ of flowers may remain closed (cleistogamous) and self fertilise while the other flowers open (chastogamous) and outcross. The zygotic genotype frequencies in the next generation are given by

$$x'_{kl} = y^{♀\prime}_{l,ij}\Omega_{f(ij)}y^{♂,p}_{k} + y^{♀\prime}_{l,ij}(1 - \Omega_{f(ij)})y^{♂,o}_{k,ij}, \tag{S1-5}$$

such that offspring produced by selfing are derived from the same individual with the same genotype. To simplify our presentation, we omit the subscript for MM females, i.e., $\Pi_{MM} = \Pi$ and $\Omega_{MM} = \Omega$. In equations (S1-4) and (S1-5), the paternally-inherited haplotype has index k and the maternally-inherited haplotype has index l. These are equivalent and are combined to get the ten zygotic allele frequencies for the next generation.

Note that gametic selection under selfing or monandry is determined by the same equation (S1-2), which sums over k gamete genotypes produced by a single male. Polyandrous matings and outcrossing allow competition between gametes from multiple males so equation (S1-3) also includes summations over all ij male genotypes. Under monandry, a female can mate with any male so equation (S1-4) sums over all ij male genotypes. On the other hand, the mother and father is the same when selfing, equation (S1-5).
