# Supplementary material for: Gametic selection favours polyandry and selfing
Source: PLoS Genet. 2024 Feb 16;20(2):e1010660. doi: 10.1371/journal.pgen.1010660 (PMC10903963; doi:10.1371/journal.pgen.1010660)
Supplement: S2 Appendix — Extrapolates one locus equilibrium allele frequencies to get genome wide mutation load and inbreeding depression. (PDF) [file pgen.1010660.s005.pdf]

S2 Appendix: Mutation Load and Inbreeding Depression

We calculate mutation load and inbreeding depression based on the equilibrium allele frequency for single loci presented in the main text. Mutation load is the reduction in mean fitness caused by deleterious mutations. Inbreeding depression, δ, is the relative reduction in fitness caused by selfing versus outcrossing, $\delta = 1 - \bar{w}_s / \bar{w}_o$ where $\bar{w}_s$ and $\bar{w}_o$ are the mean fitnesses of offspring produced by selfing and outcrossing, respectively.

We consider two sets of loci: l_B loci experience balancing selection and l_μ loci experience purifying selection (i.e., expected to be at mutation-selection balance). Following model II of Charlesworth and Charlesworth (1992), a fraction k of the l_μ loci experience purifying selection in both the diploid and male gametic phases while the remaining fraction are only expressed in diploid adults. The mean fitness across all loci is given by $\bar{w} = \bar{w}_B \bar{w}_\mu$ where $\bar{w}_B$ and $\bar{w}_\mu$ are the mean fitness effects of loci experiencing balancing selection and purifying selection, respectively.

We begin by assuming that there are no sex differences in selection and that loci of the same type have uniform effects. That is, $s_i^{♂} = s_i^{♀} = s_{i,B}$ for loci under balancing selection and $s_i^{♂} = s_i^{♀} = s_{i,\mu}$ for loci at mutation-selection balance. Given that loci are unlinked and have multiplicative fitness, the mean fitness effect of loci that experience balancing selection is given by

$$\bar{w}_B = \prod_i \left(1 + s_{i,B}\right)^{l_B z_{i,B}} \tag{S2-1}$$

where $z_{i,B}$ is the frequency of genotype i with $i \in AA, Aa, aa$. The mean fitness effect of loci at mutation-selection balance is given by

$$\bar{w}_\mu = \prod_i \left(1 + s_{i,\mu}\right)^{l_\mu \left(k z_{i,\mu} + (1-k) z_{i,\mu}^d\right)} \tag{S2-2}$$

where the frequency of genotype i at loci with and without gametic selection is

given by $z_{i,\mu}$ and $z^d_{i,\mu}$, respectively. This calculation assumes that the loci that experience gametic selection have the same fitness effect on diploid adults as those that don't experience gametic selection but this assumption can be relaxed.

Following the method of Charlesworth and Charlesworth (1992), we can approximate mutation load $(1 - \bar{w}_\mu)$ by taking sequential Taylor series for weak selection and then small mutation rates. This yields

$$\bar{w}_\mu \approx \exp\left[-l_\mu s_\mu \left(k\hat{q}_\mu + (1-k)\,\hat{q}^d_\mu\right)\right] \tag{S2-3}$$

where

$$s_\mu = (2-F)\,s_{AA,\mu} - 2\,(1-F)\,s_{Aa,\mu} - F s_{aa,\mu} \tag{S2-4}$$

gives the diploid fitness effect of deleterious alleles, $\hat{q}_\mu$ and $\hat{q}^d_\mu$ are equilibrium allele frequencies for loci that experience gametic selection (equation 2) and those that don't (equation 2 with $s^{g(i,j)}_{G(k)} = 0$). With selfing, the inbreeding coefficient is the same as in the main text, $F = (1-\Omega)\,/\,(1+\Omega)$. That is, $F = 0$ in our models of monandry versus polyandry.

Because the fitness effects of different loci are assumed to be multiplicative, the total inbreeding depression is given by

$$\delta = 1 - \delta_\mu \delta_B \tag{S2-5}$$

where the contributions of loci at mutation-selection balance and under balancing selection are

$$\begin{aligned}\delta_\mu &\approx \exp\left[l_\mu \left(k\hat{q}_\mu + (1-k)\,\hat{q}^d_\mu\right) s_{\delta_\mu}\right]\\ \delta_B &\approx 1 + l_B \hat{q}_B \left(1-\hat{q}_B\right) s_{\delta_B}\end{aligned} \tag{S2-6}$$

and the average diploid fitness effect of increased homozygosity is given by

$$
\begin{aligned}
s_{\delta_\mu} &= \left(\frac{1+F}{2}\right)\left(s_{AA,\mu} + s_{aa,\mu} - 2s_{Aa,\mu}\right), \\
s_{\delta_B} &= \left(\frac{1+F}{2}\right)\left(s_{AA,B} + s_{aa,B} - 2s_{Aa,B}\right).
\end{aligned}
\tag{S2-7}
$$

With haploid gametic expression ($s_A^{Aa} = s_A^{AA}$, $s_a^{Aa} = s_a^{aa}$) and no sex differences in selection, the growth rate of a selfing rate modifier given by equations (7) and (8) can be rewritten as $\lambda_B^\Omega \approx \lambda_\mu^\Omega \approx 1 + \Delta\Omega(1+F)\delta$, where inbreeding depression, δ, is calculated from a single locus. That is, with $k = 1$, $l_\mu = 1$, and $l_B = 0$

$$
\delta \approx \hat{q}_\mu \left(-s_{\delta_\mu}\right) = \hat{q}_\mu \frac{I'_A + I'_a}{2\Omega}
\tag{S2-8}
$$

to leading order in ϵ, and with $l_\mu = 0$, and $l_B = 1$

$$
\delta \approx \hat{q}_B \left(1 - q_B\right)(-s_{\delta_B}) = \hat{q}_B \left(1 - q_B\right) \frac{I'_A + I'_a}{2\Omega}
\tag{S2-9}
$$

where I'_A and I'_a are the invasion conditions from equation (1) with haploid gametic expression and no sex differences in selection ($s_A^{Aa} = s_A^{AA}$, $s_a^{Aa} = s_a^{aa}$, $s_i^♂ = s_i^♀ = s_{i,B}$, and $s_i^♂ = s_i^♀ = s_{i,\mu}$).

If there are sex differences in selection and/or non-haploid gametic expression, we can interpret the modifier growth rates, λ_B^Ω and λ_μ^Ω, in terms of a modified version of inbreeding depression, δ^*. To do so, we introduce a new mean fitness $\bar{w}^* = \bar{w}_B^* \bar{w}_\mu^*$ that includes gametic fitness and weights male and female fitness

terms according to the outcrossing rate, as in Table 2, such that

$$\bar{w}_B^* = \prod_i \left(\frac{\Omega\left(1 + s_{i,B}^{\male}\right)\left(1 + \bar{s}_B^i\right) + (2 - \Omega)\left(1 + s_{i,B}^{\female}\right)}{2} \right)^{l_B z_{i,B}}$$
$$\bar{w}_\mu^* = \prod_i \left(\frac{\Omega\left(1 + s_{i,\mu}^{\male}\right)\left(1 + \bar{s}_\mu^i\right) + (2 - \Omega)\left(1 + s_{i,\mu}^{\female}\right)}{2} \right)^{l_\mu z_{i,\mu}} \tag{S2-10}$$

where the average fitness of gametes produced by males with genotype i is given by $\bar{s}_B^i$ and $\bar{s}_\mu^i$ where $\bar{s}_k^{AA} = s_{A,k}^{AA}$, $\bar{s}_k^{Aa} = \left(s_{A,k}^{Aa} + s_{a,k}^{Aa}\right)/2$, and $\bar{s}_k^{aa} = s_{a,k}^{aa}$.

Calculating a modified form of inbreeding depression, δ^* to leading order in ϵ, using the weighted mean fitnesses given by equation (S2-10) gives

$$\delta^* \approx \hat{q}_\mu \frac{I_A + I_a}{2\Omega}$$
$$\delta^* \approx \hat{q}_B \left(1 - q_B\right) \frac{I_A + I_a}{2\Omega} \tag{S2-11}$$

for a single locus at mutation-selection balance ($l_\mu = 1, k = 1, l_B = 0$) or balancing selection ($l_\mu = 0$, $l_B = 1$), respectively. Thus, $\lambda_B^\Omega \approx \lambda_\mu^\Omega \approx 1 + \Delta\Omega(1 + F)\delta^*$ and a modified inbreeding depression that includes male gametic fitness and is weighted by the outcrossing rate gives a possible way of interpreting the growth rate of rare modifiers.

References

Charlesworth, D., and B. Charlesworth. 1992. The Effects of Selection in the Gametophyte Stage on Mutational Load. Evolution 46:703–720.
